# Macrophages Subvert Adaptive Immunity to Urinary Tract Infection

**DOI:** 10.1371/journal.ppat.1005044

**Published:** 2015-07-16

**Authors:** Gabriela Mora-Bau, Andrew M. Platt, Nico van Rooijen, Gwendalyn J. Randolph, Matthew L. Albert, Molly A. Ingersoll

**Affiliations:** 1 Unité d’Immunobiologie des Cellules Dendritiques, Department of Immunology, Institut Pasteur and INSERM U818, Paris, France; 2 Department of Gene and Cell Medicine and the Immunology Institute, Mount Sinai School of Medicine, New York, New York, United States of America; 3 Department of Molecular Cell Biology, Free University Medical Center, Amsterdam, The Netherlands; University of Medicine & Dentistry New Jersey, UNITED STATES

## Abstract

Urinary tract infection (UTI) is one of the most common bacterial infections with frequent recurrence being a major medical challenge. Development of effective therapies has been impeded by the lack of knowledge of events leading to adaptive immunity. Here, we establish conclusive evidence that an adaptive immune response is generated during UTI, yet this response does not establish sterilizing immunity. To investigate the underlying deficiency, we delineated the naïve bladder immune cell compartment, identifying resident macrophages as the most populous immune cell. To evaluate their impact on the establishment of adaptive immune responses following infection, we measured bacterial clearance in mice depleted of either circulating monocytes, which give rise to macrophages, or bladder resident macrophages. Surprisingly, mice depleted of resident macrophages, prior to primary infection, exhibited a nearly 2-log reduction in bacterial burden following secondary challenge compared to untreated animals. This increased bacterial clearance, in the context of a challenge infection, was dependent on lymphocytes. Macrophages were the predominant antigen presenting cell to acquire bacteria post-infection and in their absence, bacterial uptake by dendritic cells was increased almost 2-fold. These data suggest that bacterial uptake by tissue macrophages impedes development of adaptive immune responses during UTI, revealing a novel target for enhancing host responses to bacterial infection of the bladder.

## Author Summary

Urinary tract infection is a common infection with a high propensity for recurrence. The majority of infections are caused by uropathogenic *E*. *coli*, a growing public health concern with increasing prevalence of antibiotic resistant strains. Finding therapeutic options that circumvent the need for antibiotics, while boosting patients’ immune response to infection is desirable to counteract further increases in antibiotic resistance and to provide long-lasting resistance to infection. Currently, little is known about how adaptive immune responses, which typically prevent recurrent infection in other organs, arise from the bladder during urinary tract infection. Here, we investigated the initial interactions between immune cell populations of the bladder and uropathogenic *E*. *coli*, finding that macrophages are the principal cell population to engulf bacteria. Interestingly, these same cells appear to inhibit the development of adaptive immunity to the bacteria, as their depletion, prior to primary infection, results in a stronger immune response during bacterial challenge. We found that in the absence of macrophages, dendritic cells, which are the most potent initiators of adaptive immunity, are able to take up more bacteria for presentation. Our study has revealed a mechanism in which specific immune cells may act in a manner detrimental to host immunity.

## Introduction

Urinary tract infection (UTI) is one of the most common bacterial infections, impacting more than 130 million people annually worldwide [1,2]. The principal causative agent is uropathogenic *Escherichia coli* (UPEC), accounting for more than 75% of all community acquired infections, particularly among a seemingly healthy population (*e*.*g*., premenopausal women) [1]. In uncomplicated UTI (*i*.*e*., cystitis), nearly half of all women infected will experience recurrence [3]. Currently, there is little consensus in the field regarding the underlying causes of the high rate of recurrence. Mechanisms previously proposed to explain this phenomenon include that UPEC forms protected reservoirs in the bladder, remerging at later time points after initial infection [4,5]; that UPEC strains colonize the gut and periodically migrate to the urinary tract [6]; or that the immune response to infection is suppressed by mast cell-derived IL-10 in the bladder [7].

The innate immune response to UPEC infection is characterized by robust cytokine and chemokine expression, leading to rapid neutrophil and monocyte infiltration and subsequent bacterial clearance [8–12]. Depletion of both neutrophils and monocytes, by Gr1 antibody treatment [13], leads to increased bacterial burden, whereas a reduction in circulating neutrophils alone decreases bacterial burden, suggesting that monocytes help eliminate bacteria in the bladder [10,11]. A recent study demonstrated that innate immune cell crosstalk is necessary for a coordinated innate response, whereby resident macrophages, responding to signals from infiltrating monocytes, induce MMP9 expression in neutrophils, in turn facilitating their trans-urothelial migration [14]. This mechanism likely works in concert with cytokine and chemokine expression from infected urothelium that mediates neutrophil recruitment and trans-urothelial migration [9].

The mechanisms involved in the initiation of adaptive immunity, and indeed the full nature of the response generated from the bladder during UTI, remain unclear [15]. The majority of studies have focused on innate immunity to UTI, such as neutrophil or monocyte infiltration, while only a limited number of studies have focused on adaptive immune mechanisms [16]. For example, UPEC-specific antibodies arise during UTI in mice, non-human primates, and human patients, and can inhibit UPEC binding to urothelial cells *in vitro* [17–19]. With respect to the role of effector cells, only one study has examined the induction of antigen-specific antibody and T cell responses after UPEC infection, demonstrating that transfer of serum or T cells from infected animals limits infection in naïve mice [19].

In this study, we investigated the initiation of adaptive immunity to UPEC to determine whether defects exist preventing the induction of sterilizing immunity. We conclusively demonstrated that adaptive immune responses are generated in response to UPEC infection; however, they are insufficient to prevent reinfection. We performed the first systematic analysis of the tissue-resident immune cell compartment in the steady state bladder of mice and investigated the role of macrophages, and their precursors, in the adaptive immune response during UTI. Strikingly, macrophage depletion, prior to primary infection, improved adaptive immune responses to challenge infection in a macrophage-replete environment. We observed that upon infection, macrophages were the principal population, among the antigen presenting cells, to acquire UPEC early in infection, and in their absence, bacterial uptake by dendritic cells (DCs) was increased. These data support a model in which bladder-resident macrophages sequester bacteria, consequently limiting adaptive immune responses, and provides an explanation for the failure of the immune system to respond effectively to UPEC infection.

## Results

### UPEC infection primes an adaptive immune response mediated by DCs

**Fig 1:**
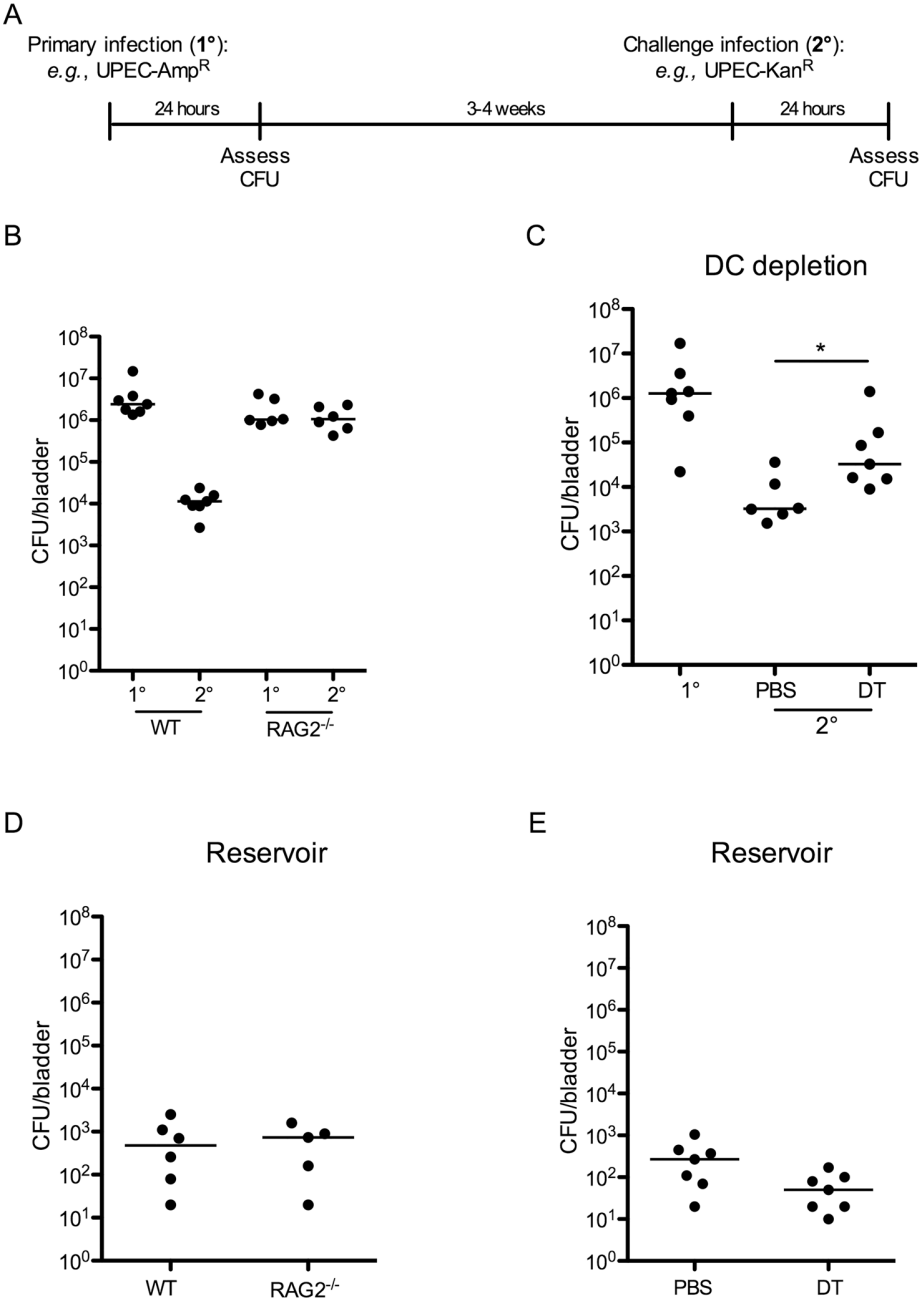
An adaptive immune response is necessary for bacterial clearance during UPEC challenge infection. (A) Experimental scheme used in the study. (B) Female C57Bl/6 (WT) or RAG2^-/-^ mice were instilled with UTI89 and sacrificed 24 hours P.I. (1°) or challenged with an isogenic UPEC strain carrying a different antibiotic marker and sacrificed 24 hours P.I. (2°) to evaluate bacterial burden. (C) 12 weeks post-reconstitution, chimeric CD11c-DTR mice were treated with PBS or diphtheria toxin (DT) to eliminate DCs and infected with UTI89 24 hours post treatment. Mice were challenged with the isogenic UPEC strain and sacrificed 24 hours P.I. to measure CFU/bladder (2°). At the time of the challenge infection in (C), an additional group of naïve CD11c-DTR chimeric mice was infected with UTI89 to evaluate CFU after a primary UPEC infection (1°). (D-E) Graphs depict CFU/bladder of the primary strain from the infections in (B) and (C), respectively. Each dot represents one mouse, lines are medians. Experiments were performed 2 times with 5–7 mice per group in each experiment. *p = 0.0221, Mann-Whitney.

Surprisingly, no study has directly tested the necessity of an adaptive immune response to limit UPEC reinfection or the role of specific components of the adaptive immune system in generating these responses. We employed a model of UPEC-induced cystitis in which 10^7^ colony-forming units (CFU) of UPEC isolate UTI89, made resistant to either ampicillin or kanamycin, were instilled intravesically into 7–8 week old female wildtype C57Bl/6 or C57Bl/6 RAG2^-/-^ mice [20]. Animals were sacrificed at 24 hours post-infection (P.I.) to assess bacterial burden or monitored for bacteriuria to evaluate the resolution of acute infection, defined by the absence of bacteria in the urine. Three to four weeks later, when the mice had resolved the primary infection, animals were challenged with 10^7^ CFU of an isogenic UPEC strain, resistant to the antibiotic not employed for primary infection, and sacrificed 24 hours P.I. to evaluate bacterial clearance (Fig 1A). Importantly, the use of isogenic UPEC strains, differing only by antibiotic resistance and fluorescent marker, permitted differentiation between quiescent bacteria residing in reservoirs established during primary UPEC infection [5] and the challenge strain. Of note, this distinction has not been made in previous reports, and thus it has remained unclear whether bacteria measured in the bladder after challenge infection derive from the primary or challenge infection, or represent a mixture of both infections [19]. After UPEC challenge in wildtype mice, we observed a >2 log reduction in CFU of the challenge UPEC strain compared to the bacterial burden 24 hours after primary infection (Fig 1B). By contrast, the bacterial burden after challenge infection in RAG2^-/-^ mice was similar to that after primary infection (Fig 1B).

As DCs are the principal cells to present antigen to lymphocytes, we investigated their role in inducing an adaptive immune response following a primary infection. Utilizing CD11c-DTR chimeric mice, we depleted CD11c-expressing DCs, prior to primary infection, by administration of two doses of diphtheria toxin (S1 Fig). As previously reported, diphtheria toxin treatment also impacted the number of tissue resident macrophages (S1 Fig and [21]); however, this reduction was minimal and the number of macrophages present in toxin-treated mice was within the range of normal variance (Table 1). Twenty-four hours after depletion, we infected mice as described in Fig 1A, and followed resolution of infection by assessing bacteriuria. Upon resolution of the primary infection, mice were challenged with an isogenic UTI89 strain, as described above. To measure the bacterial burden following primary infection in the chimeric mice, an additional cohort of naïve, untreated chimeric mice received a primary infection at the same time as the infected animals received the challenge infection (Fig 1C, 1° group). We assessed bacterial burden at 24 hours following primary or challenge infection and observed that animals treated with PBS were better able to clear UPEC after challenge compared to DC-depleted animals (Fig 1C). Together, these results suggest that the reduction in CFU observed after a challenge infection is mediated by an adaptive immune response, dependent upon DCs and lymphocytes. Interestingly, this response to UPEC reduces bacterial burden but does not prevent re-infection after challenge (Fig 1 and [19]).

**Table 1:** Immune cell populations in naïve bladders.

Naïve bladder	
Cell subset	Cell number^a^
CD45^+^	35714 ± 11161
Macrophages	9035 ± 3480
CD11b^+^ DCs	4046 ± 1785
CD103^+^ DCs	1011 ± 443
Monocytes	720 ± 467

^a^ Cell numbers are displayed as the mean ± the standard deviation. Values are derived from at least 8 mice, analyzed in separate experiments.

Finally, in our model, approximately 10% of the total CFU measured after a challenge infection arose from the primary infecting strain, representing the reservoir formed during infection. These bacteria appeared to be protected from host clearance mechanisms, as no differences in the number of bacteria present in the reservoir between wildtype and RAG2^-/-^ mice (Fig 1D), or PBS and DT-treated CD11c-DTR mice were observed (Fig 1E).

### The bladder contains a diverse immune cell repertoire

**Fig 2:**
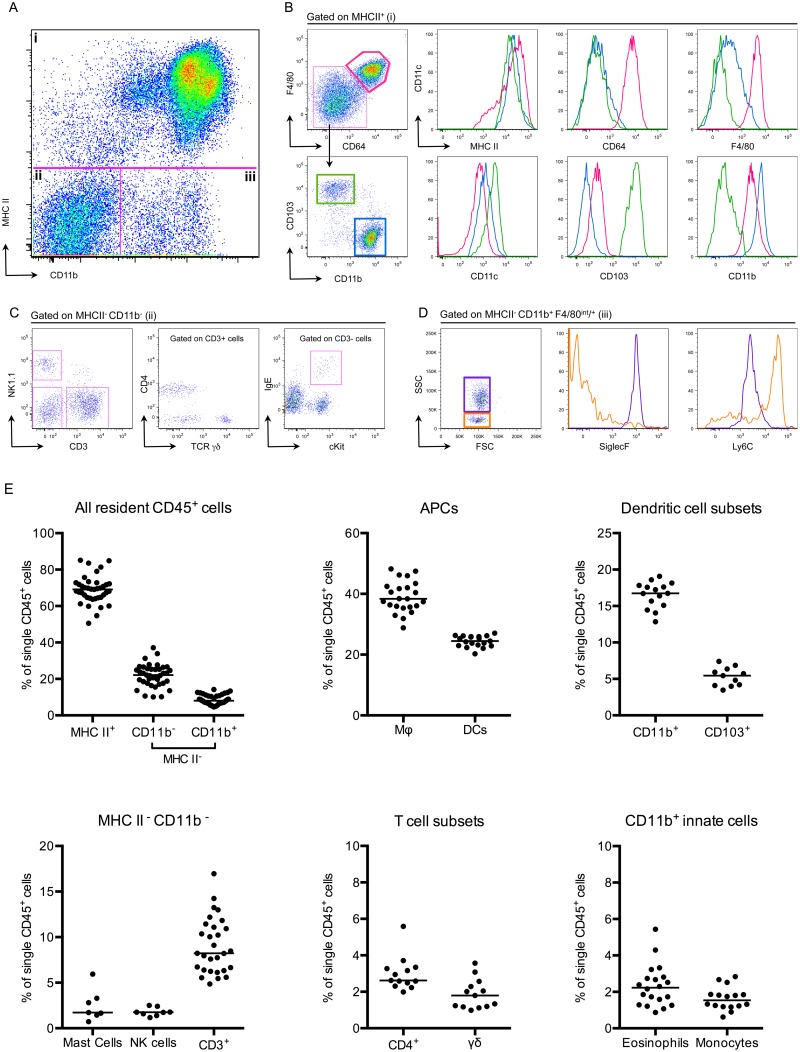
The bladder contains a diverse immune cell repertoire. Naïve bladders from female C57Bl/6 mice were digested for flow cytometry. Single cell preparations were stained with antibodies indicated in Table 2. (A) Single, CD45^+^ cells were gated into 3 groups (i-iii) according to their CD11b and MHC II expression levels. (B) MHC II^+^ cells from gate (i) were divided into 3 populations, F480^+^ CD64^+^ macrophages (pink gate), CD11b^+^ DCs (blue gate) and CD103^+^ DCs (green gate). The expression levels of MHC II, CD64, F4/80, CD11c, CD103, and CD11b are depicted in the histograms (macrophages—pink lines, CD11b^+^ DCs—blue lines, CD103^+^ DCs—green lines). (C) MHC II^-^ CD11b^-^ cells from gate (ii) were subdivided by their expression of CD3 and NK1.1. CD3^+^ cells were divided into CD4^+^ and TCR γδ^+^ and the CD3^-^ gate shows cKit^+^ IgE^+^ mast cells. (D) The dot plot, from gate (iii) depicts MHC II^-^ CD11b^+^ F480^int/+^ eosinophils (purple gate) and monocytes (orange gate). Histograms depict expression levels of SiglecF and Ly6C from gate (iii) (eosinophils—purple lines, monocytes—orange lines). (E) Graphs depict immune cell populations in the naïve bladder as percentage of all CD45^+^ cells. Representative cytometry dot plots and histograms from different individual bladders are depicted, with the exception of the mast cell plot in (C), which depicts three pooled bladders due to the low number of mast cells. In (E), each dot represents one bladder, lines are medians, plots are pooled from three or more experiments.

To understand how adaptive immune responses are initiated in the bladder, we began by investigating the resident immune cell compartment of the bladder. Earlier studies have described bladder-resident DCs [22–25], however, a comprehensive analysis of all immune cell populations has not been previously performed. Thus, we executed a systematic analysis of the bladder-resident CD45^+^ immune cell compartment. Bladders from naïve C57Bl/6 mice were enzymatically digested and immunostained (Materials and Methods, Table 2). As the bladder’s broad autofluorescent signal interfered with immune cell detection, we developed a gating strategy to exclude nonhematopoietic autofluorescent cells during analysis (S2A–S2C Fig). Antigen presenting cells (APCs), defined as MHC II^+^, comprised the majority of CD45^+^ cells (69% ± 7.5 of CD45^+^ cells, Fig 2A and 2E). Macrophages, delineated by CD64 [26,27] and F4/80 co-expression, were by far the largest APC population (~40% of CD45^+^ cells) (Fig 2B and 2E). The CD11b^+^ and CD103^+^ dendritic cell (DC) subsets represented 15% and 5% of CD45^+^ cells, respectively (Fig 2B and 2E). Within the MHC II^-^ CD11b^-^ gate, we identified NK1.1^+^ NK cells, CD11b^lo-int^ cKit^+^ IgE^+^ mast cells [28], CD3^+^ CD4^+^, and CD3^+^ γδ^+^ T cells, but never observed CD8^+^ T cells in naïve bladders (Fig 2C and 2E). Recently, it was reported that classical Ly6C^+^ monocytes constitutively traffic into naïve nonlymphoid tissues, such as skin, in the steady state [29,30]. Accordingly, in the MHC II^-^ CD11b^+^ gate, we identified resident Ly6C^+^ monocytes as well as SiglecF^+^ eosinophils [31] (Fig 2D and 2E). Notably, no neutrophils were observed in naïve bladders.

**Table 2:** Antibodies used in this study.

Molecule	Clone	Vendor
CD3	145-2C11	BD Bioscience
CD4	RM4-5	BD Bioscience
CD4	GK1.5	BD Bioscience
CD8	53–6.7	BD Bioscience
CD45	30-F11	BD Bioscience
CD64	X54-5/7.1	BD Bioscience
CD103	M290	BD Bioscience
CD115	AFS98	eBioscience
CD11b	M1/70	BD Bioscience
CD11c	N418	eBioscience
c-Kit	2B8	BD Bioscience
F4/80	CI:A3-1	AbD Serotec
gammadelta TCR	GL3	eBioscience
Gr1	RB6-8C5	BD Bioscience
IgE	R35-72	BD Bioscience
Ly6G	1A8	BD Bioscience
Ly6C	AL-21	BD Bioscience
MHC-II (I-A/I-E)	M5/114.15.2	eBioscience
NK 1.1	PK136	BD Bioscience
Nkp46	29A1.4	BD Bioscience
SiglecF	E50-2440	BD Bioscience

### Infiltrating classical monocytes differentiate to macrophages during infection

**Fig 3:**
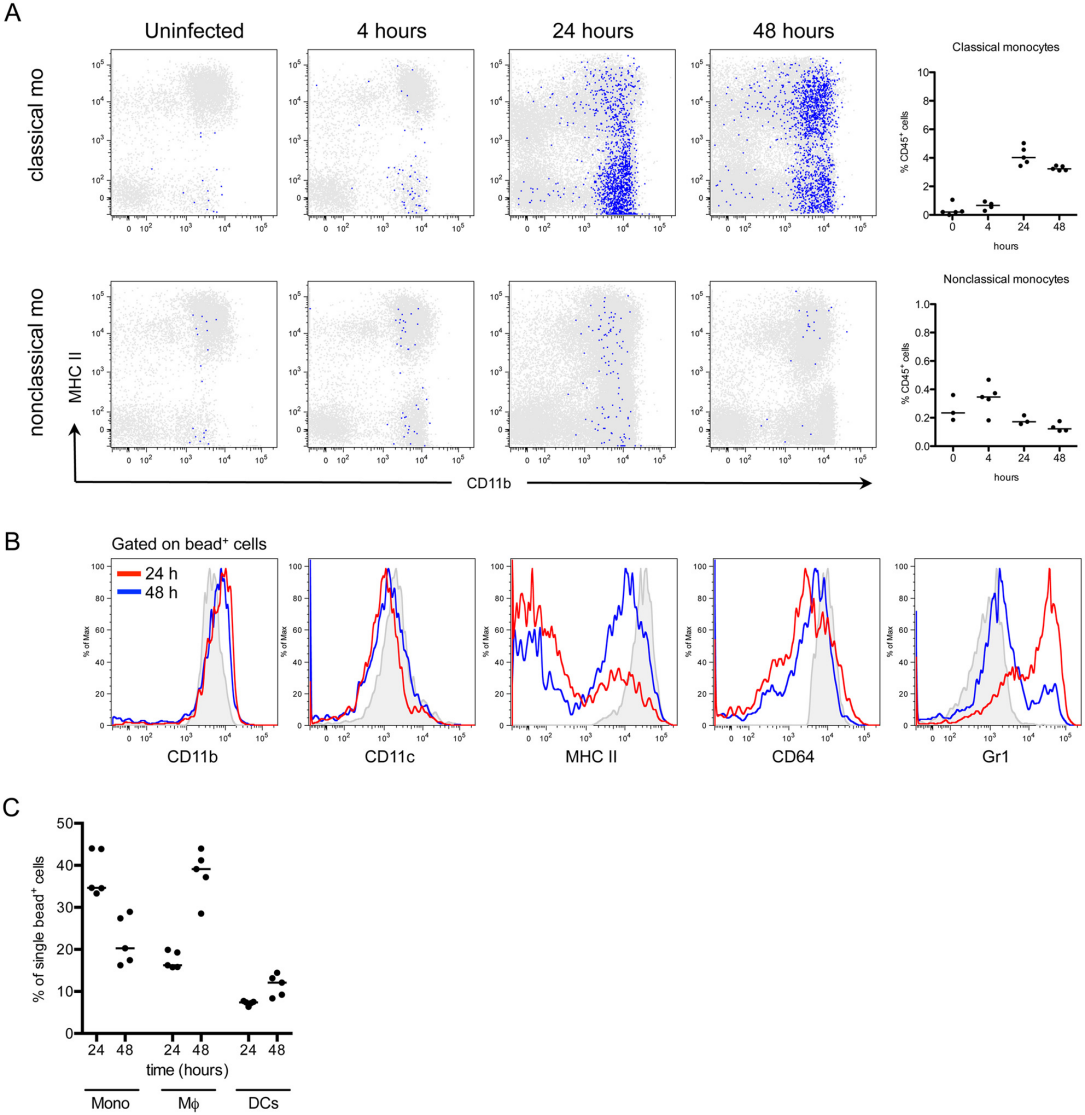
Classical monocytes robustly enter the bladder and become macrophages. In naïve C57Bl/6 mice, monocyte subsets were labeled *in vivo* as described in Materials and Methods. The designation “classical mo” and “nonclassical mo” indicates the monocyte subset labeled. Mice were infected with UTI89 24 hours after monocyte labeling and sacrificed at 4, 24, and 48 hours P.I. for flow cytometry. (A) Representative cytometry plots are shown, where gated bead^+^ cells (blue) are overlaid on all CD45^+^ bladder cells (gray). Graphs depict the quantitation of infiltrating monocytes over time, note difference in y-axis. (B) Histograms show the bead^+^ cell phenotype in the bladder over time after classical monocyte labeling (red lines—24 hours P.I., blue lines—48 hours P.I., and gray histograms—resident macrophages at 4 hours P.I., for reference). (C) Graphs show the percentage of all bead^+^ CD45^+^ cells after classical monocyte labeling in infected bladders by immune cell subset at 24 and 48 hours P.I. Each dot is one mouse, lines are medians. In (A) and (B), single representative bladders are displayed. Experiments were performed 3 times with 3–5 mice per group.

In addition to neutrophils, monocytes infiltrate the bladder upon UPEC infection [8,11]. To determine the fate of infiltrating monocytes, we employed *in vivo* labeling of circulating monocytes to monitor their entry into infected bladders [8,32]. Mice were infected 24 hours after labeling circulating classical or nonclassical monocytes and sacrificed at 4, 24, and 48 hours P.I., for analysis by flow cytometry. In line with observations from other infection models [33,34], a greater number of classical monocytes infiltrated the bladder over time than nonclassical monocytes (Fig 3A, note the scales of the y-axes). In the classical labeling protocol, a majority of infiltrated bead^+^ cells upregulated CD11c, MHC II, and CD64 expression, and downregulated CD11b and Ly6C from 24 to 48 hours P.I., phenotypically resembling resident macrophages (gray histograms) (Fig 3B). The percentage of bead^+^ cells that were identified as macrophages increased from 24 to 48 hours, while only 10% of all bead^+^ cells had a DC phenotype (Fig 3C), supporting the conclusion that infiltrating classical monocytes predominantly differentiate into macrophages during UTI.

### Depletion of bladder-resident macrophages improves adaptive immunity to UPEC

**Fig 4:**
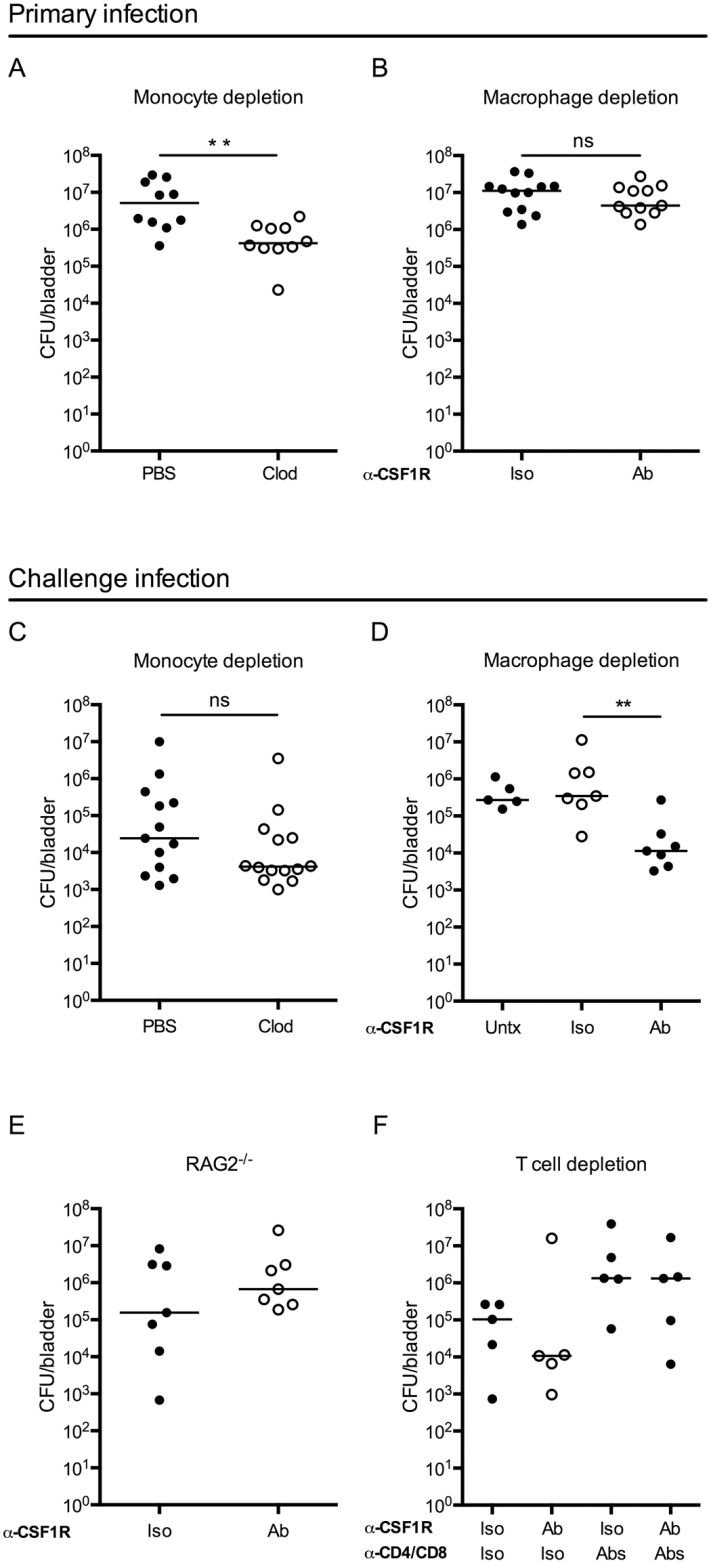
Macrophage depletion improves the adaptive response to UPEC infection. Graphs show CFU per bladder 24 hours post primary infection (A) in mice treated with PBS or clodronate liposomes (Clod) to deplete monocytes 15–18 hours prior to infection and (B) in mice treated 24 hours prior to primary infection with isotype control (Iso) or CSF1R antibody (Ab). (C-F) Mice were given a primary infection, allowed to resolve, and 3 to 4 weeks P.I., mice were challenged with an isogenic UPEC strain, as in Fig 1A. Graphs show CFU per bladder 24 hours post challenge infection in (C) wildtype mice treated with PBS or clodronate-loaded liposomes (Clod) to deplete monocytes 15–18 hours prior to primary infection, (D) isotype (Iso) or CSF1R antibody (Ab) treated mice to deplete resident macrophages 24 hours prior to primary infection, or left untreated (Untx), (E) RAG2^-/-^ mice treated with isotype (Iso) or CSF1R antibody (Ab) prior to primary infection or, (F) wildtype mice treated with isotype (Iso) or CSF1R antibody (Ab) to deplete resident macrophages and isotype (Iso) or anti-CD4 and anti-CD8 antibodies (Abs) to deplete T cells, prior to primary infection. Each dot is one mouse, lines are medians. Experiments were performed 2–4 times with 4–7 mice per group. **p = 0.0021, Mann-Whitney; ns: not significant.

During infection, infiltrating monocytes increased the already substantial macrophage compartment, thus we hypothesized that monocyte-derived and/or resident macrophages might play an important role during UPEC infection. To test this hypothesis, we depleted each population separately to determine the impact on bacterial burden. Circulating monocytes, but not bladder-resident macrophages, were depleted with clodronate liposomes [35] (S3A and S3B Fig), and mice were infected 15–18 hours later with UPEC. Mice were sacrificed 24 hours P.I. to determine CFU. Monocyte-depleted animals had a small (<1-log) but significant improvement in bacterial elimination 24 hours P.I. (Fig 4A). This difference, however, was lost if mice were infected 24 hours post-clodronate treatment, when monocytes have begun to repopulate the circulation, suggesting that monocyte depletion has a transient impact on bacterial burden. Supporting this conclusion, there were no differences in bacterial burden 24 hours P.I. in CCR2^-/-^ mice (S4 Fig), which have greatly reduced numbers of circulating monocytes [33], as compared to wildtype mice.

Bladder-resident macrophages were eliminated by administration of anti-CSF1R depleting antibody 24 hours prior to infection (S3C Fig) [36]. Importantly, the anti-CFS1R antibody also targeted monocytes, however these cells were not completely eliminated from circulation at the time of infection (compare S3A Fig to S3D Fig). Mice depleted of macrophages had a similar bacterial burden at 24 hours P.I. compared to non-depleted mice (Fig 4B) suggesting that the absence of macrophages does not impact UPEC clearance at early time points P.I.

As macrophage depletion had no impact on the primary infection, we considered whether their absence might influence the generation of an adaptive immune response. Indeed, macrophages can impact adaptive immunity via cytokine secretion or antigen sequestration. To directly test the influence of monocytes and macrophages on the generation of adaptive immunity during UTI, we depleted each of these cell types, as above. To address the role of monocytes, mice were depleted by clodronate treatment, infected, and subsequently challenged with an isogenic UTI89 strain and sacrificed at 24 hours P.I. to determine CFU, as in (Fig 1A). We did not observe a difference in bacterial burden at 24 hours post-challenge (Fig 4C) or in reservoir formation (S5 Fig), suggesting that the absence of monocytes, before primary infection, did not influence bacterial clearance after challenge infection. Further, these data demonstrate that the small improvement observed in bacterial clearance at 24 hours post-primary infection after clodronate treatment (Fig 4A), did not influence the development of an adaptive immune response to the bacteria.

To determine whether resident macrophages influence bacterial clearance after challenge, we treated mice with anti-CSF1R and infected with UPEC. Upon resolution of the primary infection, mice were challenged with an isogenic UTI89 strain and bacterial burden determined 24 hours post-challenge. In the course of the experiment, macrophage depletion did not impact reservoir formation (S5 Fig). Despite similar clearance during the primary infection, we observed a surprising reduction of nearly 2 orders of magnitude in CFU after challenge infection of mice depleted of macrophages prior to the primary infection as compared to control isotype-treated and untreated (infected and challenged, but not receiving antibody injection) mice (Fig 4D). Importantly, at the time of the challenge infection, macrophages had repopulated the bladder in depleted animals, ruling out the possibility that bacterial burden was influenced by the absence of macrophages during challenge infection (S3E Fig).

To test whether this improvement in bacterial clearance in the absence of macrophages was dependent upon components of the adaptive immune system, we depleted macrophages in RAG2^-/-^ mice. We observed no difference in the CFU per bladder after UPEC challenge between macrophage-depleted or control treated RAG2^-/-^ mice (Fig 4E). To specifically assess the necessity of T cells, we depleted macrophages in mice that had been treated with CD4 and CD8 depleting antibodies prior to primary infection. We observed fewer UPEC post-challenge only in macrophage-depleted mice that were replete of their T cells. However, mice depleted of CD4^+^ and CD8^+^ T cells did not demonstrate improved bacterial clearance after challenge independently of macrophage depletion (Fig 4F). Together, these results support the conclusion that macrophage depletion at the time of primary infection positively impacts the capacity to generate a T cell-dependent adaptive immune response against UPEC.

### Effector cell infiltration and cytokine secretion are unchanged after macrophage depletion during infection

**Fig 5:**
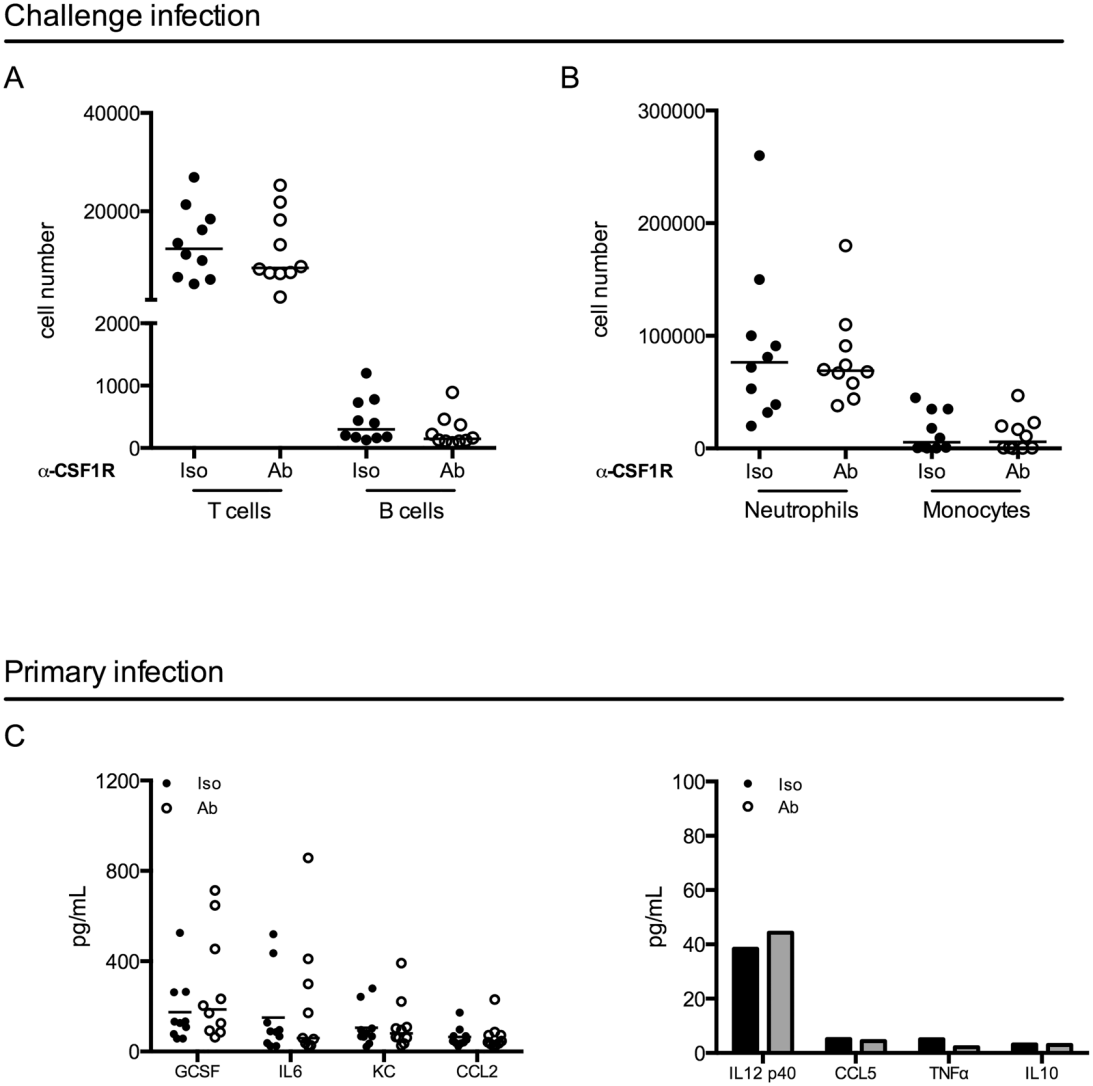
Macrophage depletion does not impact effector cell infiltration or cytokine expression. (A-B) Female C57Bl/6 mice were infected and then challenged with UPEC after resolution of their primary infection, as in Fig 1A. Twenty-four hours post-challenge, cellular infiltration into the bladder was evaluated by flow cytometry. Graphs depict cell number per bladder of the indicated populations. Experiment was repeated 3 times with 5–7 mice per group. (C) Mice were depleted with anti-CSF1R antibody and infected with 1x10^7^ CFU of UTI89 24 hours after depletion. Mice were sacrificed 24 hours P.I. and bladders were homogenized. Samples were stored at -80°C until all samples could be assessed together by Luminex multi-analyte profiling. Graphs depict the expression levels of selected cytokines in isotype antibody treated (black dots) and depleting-antibody treated (open circles) mice. Analytes are grouped by high expression (left graph) and low or no expression (right graph). Each dot represents a mouse, experiment performed 2 times with 5 mice per group and pooled, lines are medians. Additional analytes are shown in S6 Fig.

To investigate potential mechanisms mediating improved clearance after macrophage depletion in a challenge infection, we first focused on events following the challenge. We depleted macrophages and infected mice as above. When all mice had resolved the primary infection, we challenged the mice and 24 hours post-challenge, mice were sacrificed. We assessed immune cell infiltration into the bladder by flow cytometry and surprisingly, we did not observe differences in the number of T cells, B cells, neutrophils, or monocytes infiltrating the bladder after challenge infection (Fig 5A and 5B). These data suggest that improved bacterial clearance is not mediated by increased numbers of innate or effector cells. In addition to cell infiltration, we evaluated UPEC-specific IgA in the urine over time, however, the levels of UPEC-specific IgA were at the limit of detection of the assay, as has been reported in other studies [7].

Macrophages can influence the generation of an adaptive immune response through the modulation of the cytokine microenvironment [37,38]. Thus, we evaluated cytokine expression in the bladder 24 hours after a primary infection in control or antibody-treated mice by luminex technology. Notably, we did not observe significant differences between isotype-treated and antibody-depleted mice in any of the 32 cytokines evaluated (Fig 5C and S6 Fig), suggesting that cytokine expression is not significantly impacted at this timepoint in infection.

### Macrophages are the predominant APC to acquire UPEC early after infection

**Fig 6:**
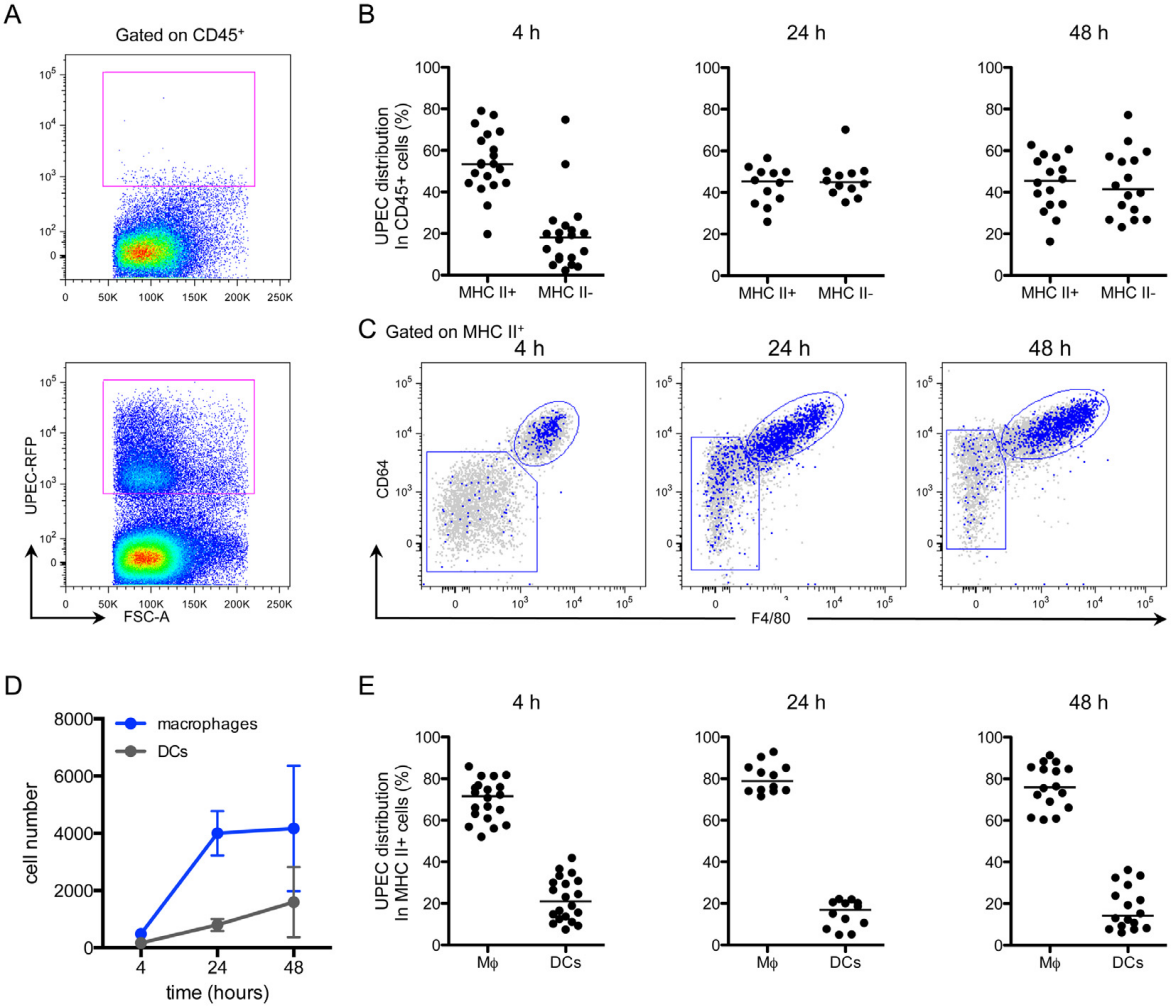
Macrophages preferentially take up UPEC at early times post-infection. Female C57Bl/6 mice were instilled with UTI89-RFP and bladders were processed for flow cytometry at 4, 24, and 48 hours. (A) Representative plots, gated on CD45^+^ cells, depict the fluorescence level of UTI89-RFP in bladders, as compared to bladders infected with the nonfluorescent parental UTI89 strain. (B) Graphs depict the distribution of UPEC between MHC II^+^ and MHC II^-^ cell populations (gated first on CD45^+^ cells) over time. (C) Representative flow plots display the distribution of bacteria in the MHC II^+^ cell populations by overlay of bacteria^+^ cells (blue) on top of all CD45^+^ cells (gray). (D) Graph depicts the number of UPEC^+^ macrophages and DCs over time. (E) Graphs show the distribution of UPEC in CD45^+^ MHC II^+^ macrophages and DCs as a percentage of UPEC acquired by all MHC II^+^ cells. In (B) and (E), each dot represents one mouse, lines are medians. In (A) and (C), single representative bladders are depicted. Experiments were performed 4 times with 4–7 mice per group and results pooled in (B), (D), (E).

Given that we found no differences in effector cell infiltration or cytokine expression, we considered whether macrophages sequester bacteria during infection. To test which immune cells acquire UPEC during infection, we utilized our kanamycin-resistant UTI89 strain, which also expresses MARS red fluorescent protein (UTI89-RFP) (S7A and S7B Fig). Importantly, when we infected mice with our ampicillin-resistant UTI89 strain expressing GFP, we could not clearly differentiate between UTI89-GFP-containing cells and the background autofluorescence of bladder macrophages (S7A Fig) or urothelial cells (S2 Fig). Importantly, UTI89-RFP had similar infectivity *in vitro* and *in vivo* compared to the parental UTI89 strain and UTI89-GFP (S7C and S7D Fig). Mice were instilled with 10^7^ CFU of UTI89-RFP and sacrificed at 4, 24, and 48 hours P.I. to analyze their bladders by flow cytometry. UTI89-RFP-containing cells were identified by gating CD45^+^ cells with RFP fluorescence levels greater than those in bladders infected with the non-fluorescent parental UTI89 strain (Fig 6A) and their phenotypes were determined based on expression of cell-specific markers as in Fig 2. At 4 hours P.I., the majority of UTI89-RFP^+^ cells were in the MHC II^+^ cell compartment. At 24 and 48 hours P.I., UTI89-RFP^+^ cells were evenly distributed between the MHC II^+^ and II^-^ gates (Fig 6B). The MHC II^-^ cells containing bacteria at 24 and 48 hours were primarily CD11b^+^ Ly6G^+^ Ly6C^+^ neutrophils and CD11b^+^ Ly6G^-^ Ly6C^+^ monocytes. Among the UPEC^+^ MHC II^+^ cells, the majority exhibited a macrophage phenotype (Fig 6C). Notably, two subpopulations were distinguishable in the macrophage gate at 24 and 48 hours P.I., representing resident (CD64^hi^ F4/80^hi^ Ly6C^-^) and monocyte-derived macrophages (CD64^int^ F4/80^int^ Ly6C^+^) (Fig 6C). Indeed, while the number of DCs harboring UTI89-RFP changed very little, the number of macrophages containing bacteria increased more than 7-fold at 24 hours and remained elevated at 48 hours (Fig 6D). At all timepoints analyzed, macrophages harbored approximately 60–80% of the bacteria found within the MHC II^+^ APC compartment, demonstrating that macrophages were the primary cell type to phagocytose bacteria at early timepoints post-infection (Fig 6E).

### Macrophage depletion leads to increased phagocytosis of UPEC by DCs

Macrophages can influence the generation of an adaptive immune response by antigen sequestration, as has been observed in the lung, hindering antigen presentation and subsequent T cell priming by DCs [39–42]. More specifically, it has been proposed that DC migration and the induction of adaptive immunity can only occur in the lung after the phagocytic capacity of macrophages has been saturated and excess antigen is available to DCs [42,43]. To test the hypothesis that antigen availability during primary infection impacted initiation of an adaptive response during UTI, we infected untreated mice with 10^7^, 10^8^, or 10^9^ CFU of UPEC. When the animals had resolved the infection, they were challenged with 10^7^ CFU of an isogenic UPEC strain and 24 hours post-challenge, sacrificed to determine bacterial burden. Notably, we did not observe a difference in the number of bacteria per bladder after a primary challenge despite increasing the inoculum 100-fold (S8A Fig). Furthermore, we did not observe any differences in bacterial burden after challenge among the three groups (S8B Fig). Thus, increasing the number of bacteria during primary infection did not improve the generation of an adaptive immune response during UTI, however it is possible that we did not saturate bladder resident macrophages with bacteria.

**Fig 7:**
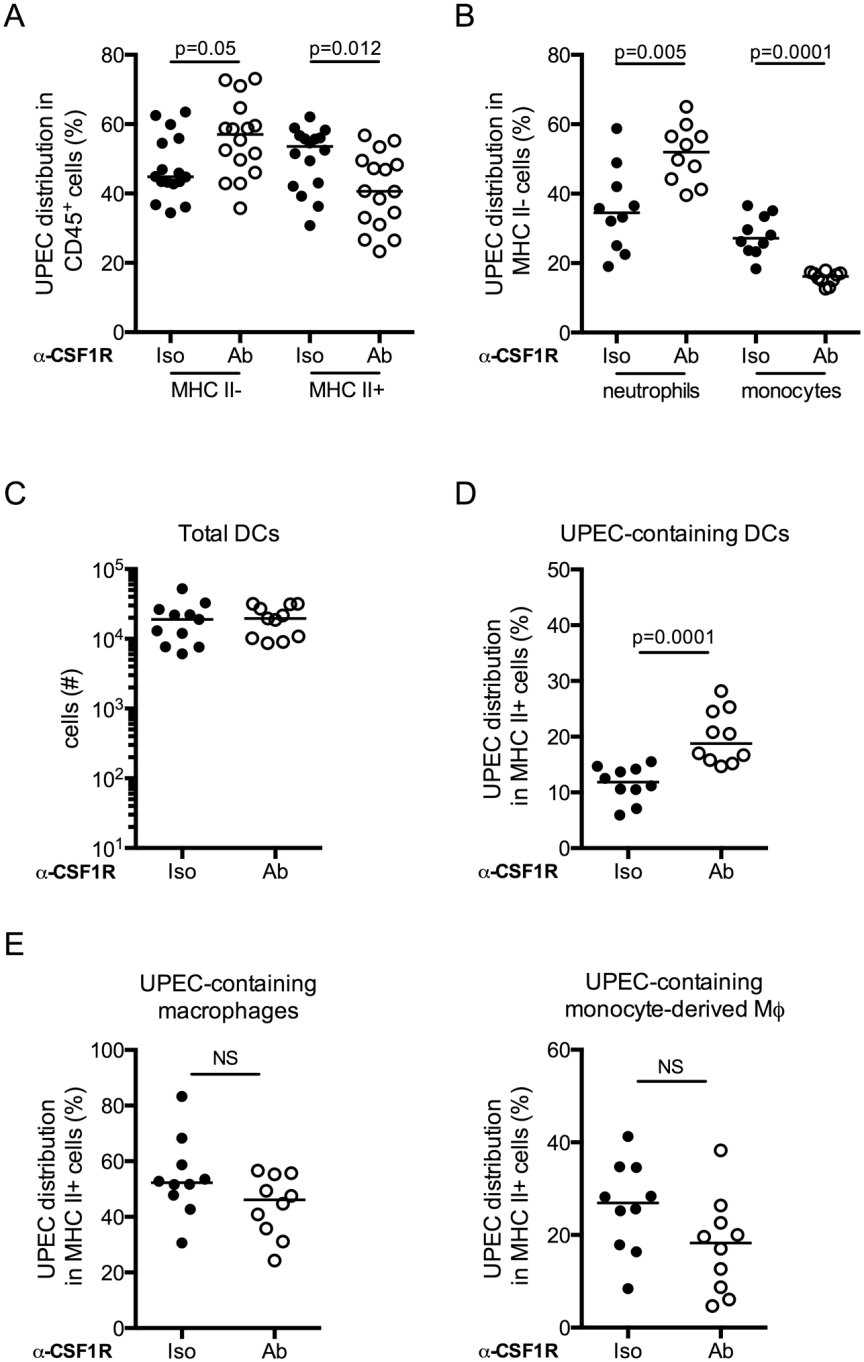
Dendritic cells acquire more bacteria in the absence of macrophages. Macrophages were depleted or not and mice were infected. Twenty-four hours post-primary infection, bladders were analyzed by flow cytometry to determine distribution of UPEC in (A) the MHC II^-^ and MHC II^+^ compartments, (B) neutrophils and monocytes. (C) Plot depicts the total number of DCs present in the bladder 24 hours post-primary infection. (D-E) Plots depict the percentage distribution of UPEC in the MHC II^+^ compartment by cell population (D) DCs and (E) resident macrophages and monocyte-derived macrophages. Each dot represents one mouse, lines are medians. Experiments were repeated 3 times with 4–7 mice per group in each experiment and results pooled. p-values indicated on graphs, Mann-Whitney test.

To directly test whether macrophages were physically sequestering UPEC in the bladder during UTI, we evaluated bacteria uptake in the bladder in the context of macrophage depletion. Mice were depleted or not of macrophages by anti-CSF1R antibody, infected with UTI89-RFP and sacrificed 24 hours P.I. The distribution of UPEC was altered in mice depleted of macrophages as compared to the control group. More bacteria localized to MHC II^-^ cells (Fig 7A and 7B), potentially explaining why macrophage depletion did not impact bacterial clearance after primary infection. The percentage of neutrophils containing bacteria was specifically increased in macrophage-depleted mice, likely compensating for the lack of monocytes and resident macrophages (Fig 7B). In MHC II^+^ cell populations, while the total number of DCs in infected bladders was unchanged (Fig 7C), we observed a significantly greater percentage of DCs had taken up UPEC in macrophage-depleted animals (Fig 7D). Notably, the percentage of UPEC-containing resident and MHC II^+^ monocyte-derived macrophages was not different between the isotype and depleting antibody groups (Fig 7E).

## Discussion

UTI is unusual in that it is a common infection that recurs with high frequency, particularly in otherwise healthy adult women [1], suggesting a defect exists in the ability to mount an adaptive immune response to UPEC. We observed that the absence of B and/or T cells or DCs impaired the host’s capacity to clear bacteria after a challenge infection, confirming that adaptive immune responses are primed during UTI. Although this was an expected result, surprisingly, it has never been formally demonstrated in the literature until now. We further demonstrated that while immune responses are primed, they neither prevent reinfection nor eliminate the bacterial reservoir established during primary infection. To shed light on potential mechanisms preventing the development of effective adaptive immunity to UPEC infection, we focused on the role of MHC II^+^ cells as they are the key initiators of adaptive immunity. Unexpectedly, in the context of a challenge infection, resident macrophage depletion improved the host’s ability to eliminate bacterial load. Importantly, macrophages were depleted prior to the first infection; however, they were present in normal numbers at the time of challenge infection. Notably, this improvement was dependent on the adaptive immune system as the phenotype was lost when macrophages were depleted in RAG2^-/-^ mice or in mice depleted of T cells. Given that macrophages were the principal APC to acquire UPEC early in infection, these data suggest that macrophages subvert initiation of a robust adaptive immune response during UTI.

To understand the mechanism of macrophage subversion of adaptive immunity during UTI, we evaluated events post-challenge and post-primary infection. We observed a significant infiltration of T cells and a smaller infiltration of B cells post-challenge but no major differences in cell numbers between the control and treated groups. At this time, we cannot rule out potential qualitative differences in the activation or specificity of the infiltrating effector cells, including T cells or possibly NK T cells, which may play a role in kidney infection [44]. As macrophages repopulated the bladder before challenge infection, we hypothesized that the impact on adaptive immunity occurred in the first few hours or days following primary infection. To explore the possibility of antigen sequestration, we evaluated which immune cell populations acquired UPEC in the bladder in the absence of macrophages. We observed an increase in the percentage of DCs containing bacteria. DCs are key players in initiating adaptive immune responses, and we found that they can do so from the bladder mucosa in the context of UTI. Indeed, even a partial depletion of bladder-resident DCs, prior to primary infection, rendered animals less capable of clearing bacteria after challenge infection. The intermediate clearance phenotype observed in DT-treated mice may have been mediated by DCs remaining after depletion or by DCs repopulating the tissues before the primary infection was resolved, permitting delayed antigen presentation. Thus, our data suggest that the more efficacious adaptive immune response against UPEC observed during macrophage depletion may be mediated by the increase in the percentage and number of DCs carrying bacteria. Notably, however, the proportion of DCs containing UPEC in infected bladders was less than 5% of total DCs present in mice, suggesting that only a very small number of antigen-carrying DCs are required to mount an adaptive immune response during UTI.

Macrophages outnumbered DCs in both naïve and infected bladders and were the principal cell to acquire UPEC. Our findings contradict a recent study in which Schiwon *et al*. suggest that bladder-resident macrophages sense UPEC infection, but do not phagocytose bacteria [14]. As an explanation for this apparent discrepancy, we found that MHC II^+^ cells containing GFP-expressing bacteria were indistinguishable from autofluorescent but uninfected cells. We engineered UTI89 to express a red fluorescent protein to specifically overcome the challenge of distinguishing naturally autofluorescent cells in the bladder (*e*.*g*., macrophages and urothelial cells) from those containing UPEC. Lending credence to this interpretation, the authors also did not detect GFP-expressing UPEC in urothelial cells [14], which are invaded during the course of UTI, as their autofluorescence also likely masked the GFP signal [45,46].

We evaluated macrophages because of their prominent role in bacterial acquisition. However, even when the majority of UPEC was captured by macrophages at early timepoints P.I., their depletion did not impact bacterial clearance after the primary infection. This apparent contradiction may be explained by the increased bacterial uptake by neutrophils observed in the absence of macrophages. Our data support a model in which macrophages sequester bacteria from DCs early in infection, however we cannot rule out that depletion of macrophages alters the microenvironment during infection, despite our negative findings in bladder homogenates. Indeed, a recent study suggests that IL-10 expression from mast cells suppresses adaptive immunity to UPEC [7]. However, in the course of our study, we found few mast cells in naïve bladder tissue. Furthermore, multi-analyte cytokine analysis revealed no striking differences between control and depleted mice and we could not detect IL-10 expression in this or a prior study [11]. The reasons for this are unclear, however may be due to the significant variation that exists in the genomes of commonly used strains such as cystitis strain UTI89, pyelonephritis strains J96, 563, CFT073, and clinical isolates (see phylogenetic tree in [47]).

Having defined an early role for resident macrophages and DCs during UTI, our work significantly advances the understanding of how adaptive responses to UPEC are achieved. However, we still do not completely understand how the adaptive immune system eliminates UPEC. Though we were not able to detect UPEC-specific antibodies above the limit of detection of our assay, others have identified that antibodies against the bacteria are generated during infection [17–19]. Thus, we hypothesize that the protection induced during UTI following a primary infection is mediated by an antibody response; however T cells may also play a critical role in the killing of infected cells. With respect to the role of the bladder macrophage, our data point to a barrier in the immune system that must be overcome, particularly for patients with recurrent UTI. Although macrophage sequestration of particulate antigen in the lung has been described, this is, to the best of our knowledge, the first study to propose a role for the physical sequestration of antigen during live bacterial infection. Strategies that increase DC number or migration may overcome the subversion imposed by macrophages, providing a viable solution to treat patients with recurrent UTI.

## Materials and Methods

### Ethics statement

At Mount Sinai School of Medicine, mouse experiments were conducted in accordance with approval of protocol number LA11-00003 by the Institutional Animal Care and Use Committee at Mount Sinai School of Medicine, which adheres to the guidelines put forth by the Animal Welfare Act and the Public Health Service policy on Humane Care and Use of Laboratory Animals. At Institut Pasteur, mouse experiments were conducted in accordance with approval of protocol number 2012–0024 by the Comité d’éthique en expérimentation animale Paris Centre et Sud (the ethics committee for animal experimentation), in application of the European Directive 2010/63 EU. In our experiments, mice were anesthetized either by inhalation of isoflurance (3–4%) or by injection of 100 mg/kg ketamine and 5 mg/kg xylazine and sacrificed either by cervical dislocation or carbon dioxide inhalation.

### Bacterial strains

The human UPEC cystitis isolate, UTI89 (kind gift from Scott Hultgren) [46], and the fluorescent protein-expressing strains UTI89-RFP and UTI89-GFP were used for infection. Briefly, fluorescent bacteria were engineered using lambda red recombination [48] to introduce an *aphA-marsRFP* or *bla-GFP* cassette in the UTI89 chromosome at the *attB* lambda phage integration site. UTI89 is sensitive to antibiotics, UTI89-RFP is resistant to kanamycin, and UTI89-GFP is resistant to ampicillin. Bacteria were grown overnight in static cultures at 37°C in Luria-Bertani broth (LB) in the presence of antibiotics (kanamycin 50 μg/mL or ampicillin 100 μg/mL) where appropriate.

### Cell lines and *in vitro* invasion assay

The mouse urothelial cell line NUC-1 [49] was used to evaluate the *in vitro* invasion efficacy of each UTI89 strain. Fifty thousand cells were infected with UTI89, UTI89-GFP, or UTI89-RFP at increasing MOIs. Thirty minutes post-infection, cells were washed, lysed, and serial dilutions were plated. Percent invasion was calculated by dividing the number of bacteria inside the cells by the inoculum x 100.

### Mice and infections

Female C57BL/6 mice between 6 and 8 weeks old were from The Jackson Laboratory or Charles River. CD11c-DTR mice were a kind gift from Marc Lecuit and Claude LeClerc (Institut Pasteur). RAG2^-/-^ mice were a kind gift from Antonio Freitas (Institut Pasteur). Briefly, mice anesthetized with isoflurance (4%) or 100 mg/kg ketamine and 5 mg/kg xylazine were infected with 10^7^ colony-forming units (CFU) of one of two UTI89 strains in 50 μL PBS via a catheter introduced into the urethra [20] except in the inoculum escalating experiment, where mice received 10^7^, 10^8^ or 10^9^ CFU in 50 μL PBS. To calculate CFU, bladders were aseptically removed and homogenized in 1 mL PBS. Serial dilutions were plated on LB agar, with or without antibiotics, as required. For challenge infection experiments, mice were infected with one of the two fluorescent strains of UTI89, expressing antibiotic resistance (kanamycin or ampicillin) (See Fig 1A). Once the primary infection cleared, 3 to 4 weeks, mice were infected with 10^7^ CFU of an isogenic UTI89 strain with a different antibiotic resistance. The strain used for the challenge infection was determined by that used in the primary infection, such that the antibiotic resistances were different between the primary and challenge infection, *e*.*g*., UTI89-GFP for the primary and UTI89-RFP for the challenge infection. Importantly, both strains were used as the primary or the challenge strain in different experiments. Resolution of infection was monitored by plating urine every 5–6 days on antibiotic-containing plates.

### Irradiation, bone marrow cell transfer, diphtheria toxin treatment

C57Bl/6 mice were irradiated with a single dose of 5–6 gray in an x-ray irradiator at 6 weeks of age. Animals were reconstituted with 1.6–3.2 x 10^6^ total bone marrow cells from CD11c-DTR mice 6 hours after irradiation. Mice were allowed to reconstitute for a minimum of 12 weeks and reconstitution was evaluated by flow cytometry of congenic markers. 24 and 48 hours prior to infection, mice were administered 4 ng/g of diphtheria toxin I.V. Depletion efficiency was tested in each batch of chimeric mice prior to experimentation.

### Flow cytometry of bladder tissue

At indicated timepoints, bladders were removed and minced with dissection scissors into tubes containing digestion buffer kept at 4°C. Minced tissue was then incubated at 37°C in 1 mL of digestion buffer containing 0.34 U/mL of Liberase TM (Roche) and 100 μg/mL of DNase in PBS. Tubes were vigorously shaken by hand every 15 minutes. 45 minutes to one hour post-incubation, when the tissue had a glassy, transparent appearance and was almost entirely digested, digestion was stopped by adding several mL of PBS supplemented with 2% FBS and 0.2 μM EDTA. The entire bladder digest was passed through a 100 μM cell strainer to obtain a single cell suspension. Gentle pressure was applied to any tissue remaining in the strainer. Samples were washed, Fc receptors blocked, and stained with antibodies listed in Table 2. Total cell counts in the bladder were determined by the addition of AccuCheck Counting beads (Invitrogen) to a known volume of sample after staining, just prior to cytometer acquisition. Gating strategies for all cell populations except for neutrophils are depicted in Fig 2. Neutrophils were identified as MHC II^-^, CD11b^+^, Ly6G^+^, Ly6C^-^, SiglecF^-^, and F4/80^-^.

### Flow cytometry of blood

To identify cell populations in the circulation, whole blood was incubated with BD PharmLyse, (BD Bioscience) and subsequently stained with antibodies indicated in the Table 2. Samples were acquired on a BD LSRFortessa using DIVA software and data were analyzed by FlowJo (Treestar) software. Total cell counts in the blood were determined by the addition of AccuCheck Counting beads (Invitrogen) to 10 μL of whole blood diluted in 1-step Fix/Lyse Solution (eBioscience).

### Monocyte bead labeling

*In vivo* bead labeling of classical and nonclassical monocytes was performed as previously described [32]. Briefly, classical monocytes were labeled by I.V. administration of 200 μL clodronate liposomes to transiently deplete all monocytes and then by I.V. injection of 1 μM nondegradable fluorescent particles 24 hours later. Nonclassical monocytes were labeled by injection of 1 μM nondegradable fluorescent particles without prior monocyte depletion. Labeling efficiency was confirmed by flow cytometry.

### Immune cell depletion

To deplete monocytes, wildtype C57BL/6 mice received I.V. injection of 200 μL of clodronate liposomes (or PBS control liposomes) 15–18 hours prior to infection [35]. Anti-CSF1R antibody (2 mg/mL, clone AFS98, eBioscience) was used to deplete bladder-resident macrophages. Animals received two I.V. injections, on consecutive days, of anti-CSF1R antibody or isotype control (clone eBR2a, eBioscience). We administered 400 μg/mouse on day 1 and 200 μg/mouse on day 2, to decrease the impact on circulating monocytes. To deplete T cells, 100 μg of CD4 (clone GK1.5, Bio X Cell) and 100 μg of CD8 (clone YTS 169.4, Bio X Cell) per mouse were injected together intraperitoneally 24 hours prior to primary infection. 200 μg of isotype control (clone LTF-2, Bio X Cell) per mouse was injected intraperitoneally. The depletion was repeated 5 days post-infection, and once a week to maintain the depletion until challenge infection.

### Luminex MAP analysis

Mice were infected with UTI89 and bladders removed 24 hours P.I. Bladders were homogenized with a handheld tissue grinder in 1 mL PBS on ice. After removal of a 100 μL aliquot to calculate CFU by serial dilution, bladder homogenates were clarified by microcentrifugation (13K, 4, 5 minutes) and stored at -80°C until assessment by Luminex Milliplex MAP Mouse Cytokine/Chemokine Magnetic Bead Panel, Premixed 32-Plex, according to the manufacturer’s recommendations (Merck Millipore) [11]. All samples were assessed together to avoid inter-assay variability. Just prior to analysis, after thawing, samples were centrifuged a second time to remove any cell debris.

### Statistical analysis

GraphPad Prism was used to evaluate statistical significant. Graphs depict medians and statistical significance was determined by the nonparametric Mann-Whitney test.

## Supporting Information

S1 Fig DT-mediated DC ablation. Irradiated C57Bl/6 mice were reconstituted with bone marrow from CD11c-DTR animals and allowed to rest for 12 weeks. Prior to infection, mice were treated two times with PBS (-) or 4 ng/g diphtheria toxin (+). 24 hours post-treatment, a cohort of animals were analyzed by flow cytometry to assess the extent of depletion in the bladder. Graph depicts the number of DCs in the bladder in PBS or DT treated mice. Each dot is one mouse, lines are medians. Depletion efficiency was tested in each batch of chimeric mice prior to experimentation, n = 2–4 mice per group.
(TIF)

S2 Fig Bladder autofluorescence. (A) Gating strategy for whole bladder digests. Bladders from naïve mice were processed as described in Materials and Methods. The entire bladder preparation was acquired and live cells were gated based on their forward and side scatter properties (top left). CD45^+^ cells were identified (top, middle), however, this population contained a large contaminating cell population (gated in pink, top right), particularly when CD45 was conjugated to fluorophores that emit near the emission wavelength of GFP. To eliminate the contaminating autofluorescent cells from our analyses, we selected single cells (FSC-W, SSC-W) versus MHC II staining (bottom, left, middle). The autofluorescent population was reduced by this strategy while immune cell populations remained (bottom, right) (B) Micrograph of the luminal surface of an *en face* whole mount prepared bladder stained only with DAPI (blue) to reveal DNA, to illustrate the intrinsic autofluorescence in this tissue. (C) Graphs depict the percentage decrease in the contaminating cell population (left) and the relative change in the myeloid cell populations in the bladder after each gating step (right, CD11b^+^ cells are derived from black gates and CD11c^+^ cells are derived from blue gates in (A), and contaminating cells are gated in pink).
(TIF)

S3 Fig Immune cell ablation. (A-B) Mice were treated with PBS or clodronate liposomes (Clod) I.V. and 15–18 hours later, blood and bladder samples were obtained to evaluate immune cell depletion. Graphs depict the percentage of (A) monocytes and neutrophils in blood and (B) monocytes, macrophages, and DCs in the bladder after treatment. (C-D) Mice received two injections of anti-CSF1R antibody (Ab) or control isotype antibody (Iso) and 24 hours post-treatment, naive bladders were isolated to evaluate immune cell depletion. Graphs show the (C) percentage and cell number of macrophages and DCs in the bladder and (D) percentage of monocytes and neutrophils in the blood. (E) Mice were depleted of macrophages as in (C-D), however, bladders were evaluated for repopulation by macrophages 4 weeks after depletion, prior to challenge infection in additional cohorts of treated mice. Each dot represents one mouse. Experiments were repeated 2–4 times with 2–7 mice per group.
(TIF)

S4 Fig CCR2^-/-^ mice are not impaired in bacterial clearance after primary infection. Graph depicts the CFU/bladder 24 hours post-primary infection in wildtype (WT) or CCR2^-/-^ mice. Experiment was repeated 2 times with 4–5 mice per group.
(TIFF)

S5 Fig UPEC reservoirs are not altered in monocyte or macrophage depleted mice. Graphs depict CFU/bladder arising from the primary infecting strain in an experiment in which (A) monocytes or (B) macrophages were depleted prior to primary infection and then challenged with an isogenic strain and sacrificed 24 hours post-challenge. Each dot represents one mouse. Experiments were repeated 2–4 times with 2–7 mice per group.
(TIFF)

S6 Fig Macrophage depletion does not impact cytokine expression post-primary infection. Mice were depleted with anti-CSF1R antibody and infected with 1x10^7^ CFU of UTI89 24 hours after depletion. Mice were sacrificed 24 hours P.I. and bladders were homogenized. Samples were stored at -80°C until all samples could be assessed together by Luminex multi-analyte profiling, to avoid inter-assay variability. Graphs depict the expression levels of selected cytokines in isotype antibody treated (black dots, red medians) and depleting-antibody treated (open circles, blue medians) mice. Analytes are grouped by high expression (top) to low or no expression (bottom). Each dot represents a mouse, experiment performed 2 times with 5 mice per group and all data pooled.
(TIFF)

S7 Fig Fluorescent UPEC strains. (A) Cytometry plots, gated on all CD45^+^ cells, depict GFP fluorescence (gated in pink with percentages) in mice either uninfected or infected with UTI89-GFP at 4 hours post-infection. (B) Fluorescence of UTI89-GFP and UTI89-marsRFP was confirmed by microscopy. (C) The mouse urothelial cell line, NUC-1, was infected with the parental UTI89, UTI89-GFP, or UTI89-RFP at an MOI of 1,10, or 100. Cells were lysed and bacterial titers determined by serial dilution 30 minutes P.I. The percentage of invasion refers to the number of bacteria obtained after infection x 100/number of bacteria in the inoculum. (D) Mice were instilled with 1x10^7^ CFU of UTI89, UTI89-GFP, or UTI89-RFP. CFU per bladder were determined by serial dilution at 24 h P.I. Each dot represents one mouse. Experiments were repeated 2 times.
(TIF)

S8 Fig Increasing bacterial inoculum in a primary infection does not improve the response to challenge infection. Female C57Bl/6 mice were instilled with 1x10^7^, 1x10^8^, or 1x10^9^ CFU of UPEC and challenged with 1x10^7^ CFU after resolution of the primary infection as in Fig 1A. (A) Plot depicts CFU 24 hours post-primary infection. (B) Graph shows CFU 24 hours post-challenge. Each dot represents one mouse. Experiments were repeated 2 times with 5–7 mice per group.
(TIFF)
