# Supplementary figures and images for: Macrophages Subvert Adaptive Immunity to Urinary Tract Infection
Source: PLoS Pathog. 2015 Jul 16;11(7):e1005044. doi: 10.1371/journal.ppat.1005044 (PMC4504509; doi:10.1371/journal.ppat.1005044)

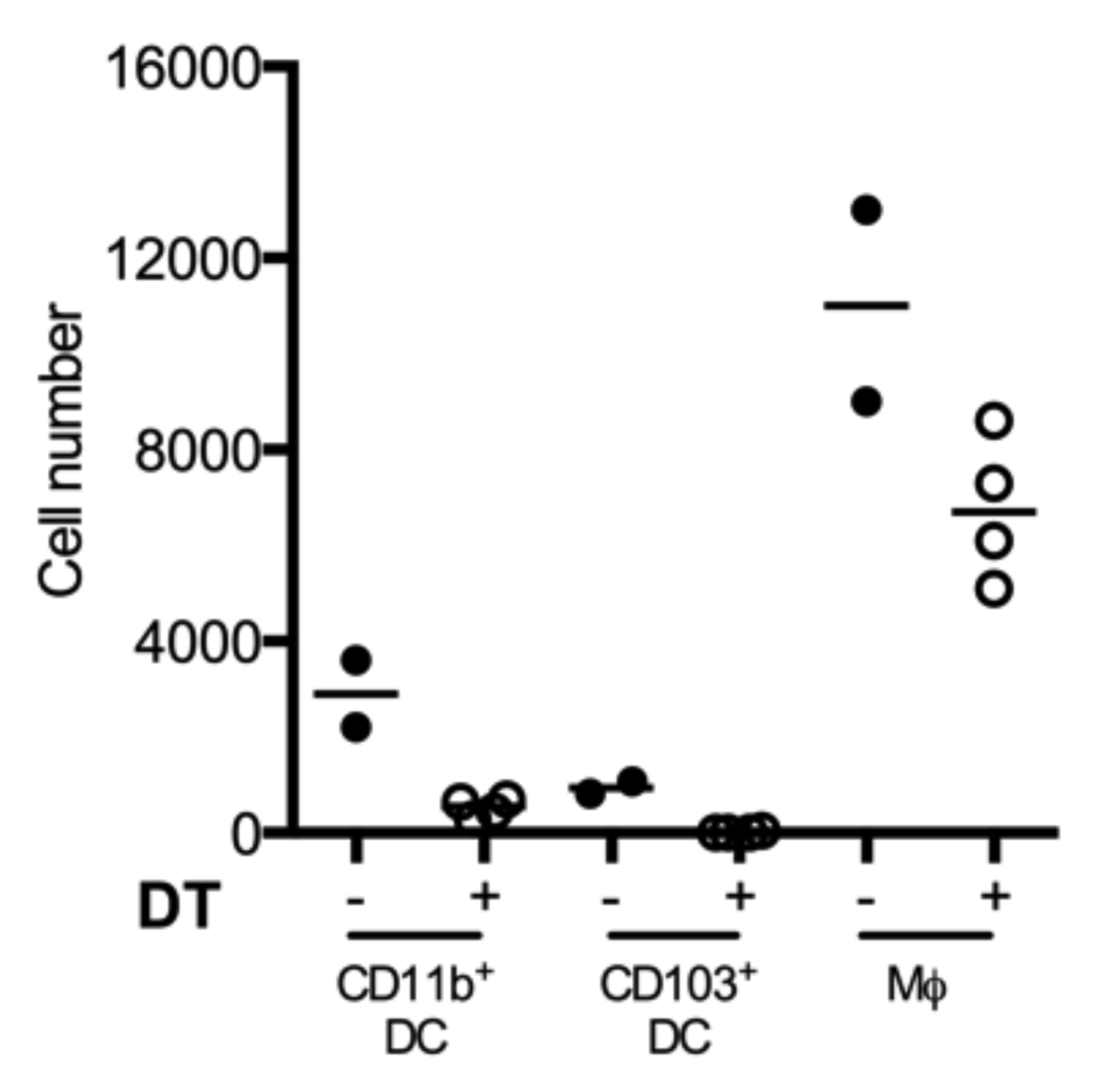

Supplement: S1 Fig — Irradiated C57Bl/6 mice were reconstituted with bone marrow from CD11c-DTR animals and allowed to rest for 12 weeks. Prior to infection, mice were treated two times with PBS (-) or 4 ng/g diphtheria toxin (+). 24 hours post-treatment, a cohort of animals were analyzed by flow cytometry to assess the extent of depletion in the bladder. Graph depicts the number of DCs in the bladder in PBS or DT treated mice. Each dot is one mouse, lines are medians. Depletion efficiency was tested in each batch of chimeric mice prior to experimentation, n = 2–4 mice per group. (TIF) [file ppat.1005044.s001.tif]

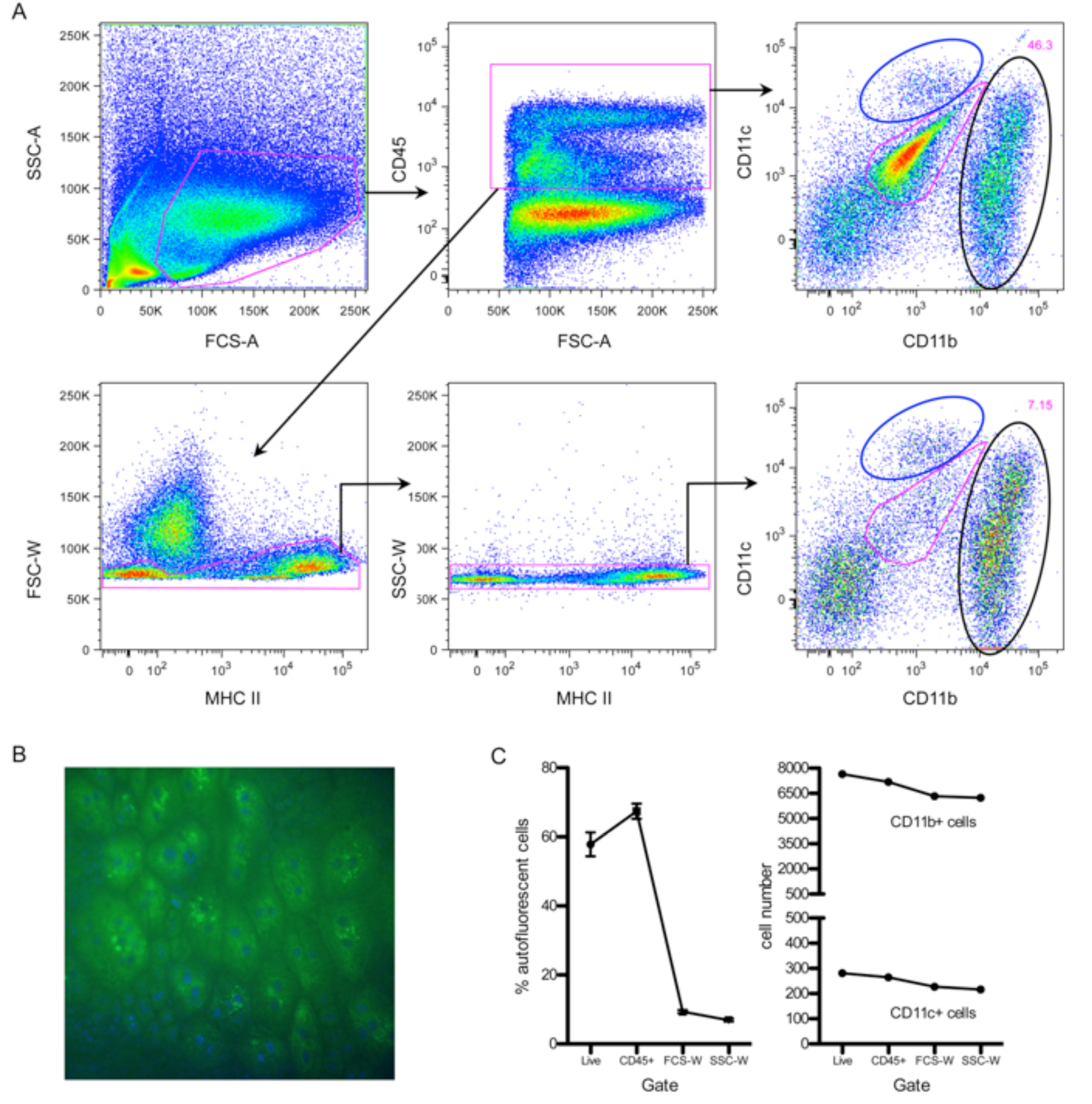

Supplement: S2 Fig — (A) Gating strategy for whole bladder digests. Bladders from naïve mice were processed as described in Materials and Methods. The entire bladder preparation was acquired and live cells were gated based on their forward and side scatter properties (top left). CD45+ cells were identified (top, middle), however, this population contained a large contaminating cell population (gated in pink, top right), particularly when CD45 was conjugated to fluorophores that emit near the emission wavelength of GFP. To eliminate the contaminating autofluorescent cells from our analyses, we selected single cells (FSC-W, SSC-W) versus MHC II staining (bottom, left, middle). The autofluorescent population was reduced by this strategy while immune cell populations remained (bottom, right) (B) Micrograph of the luminal surface of an en face whole mount prepared bladder stained only with DAPI (blue) to reveal DNA, to illustrate the intrinsic autofluorescence in this tissue. (C) Graphs depict the percentage decrease in the contaminating cell population (left) and the relative change in the myeloid cell populations in the bladder after each gating step (right, CD11b+ cells are derived from black gates and CD11c+ cells are derived from blue gates in (A), and contaminating cells are gated in pink). (TIF) [file ppat.1005044.s002.tif]

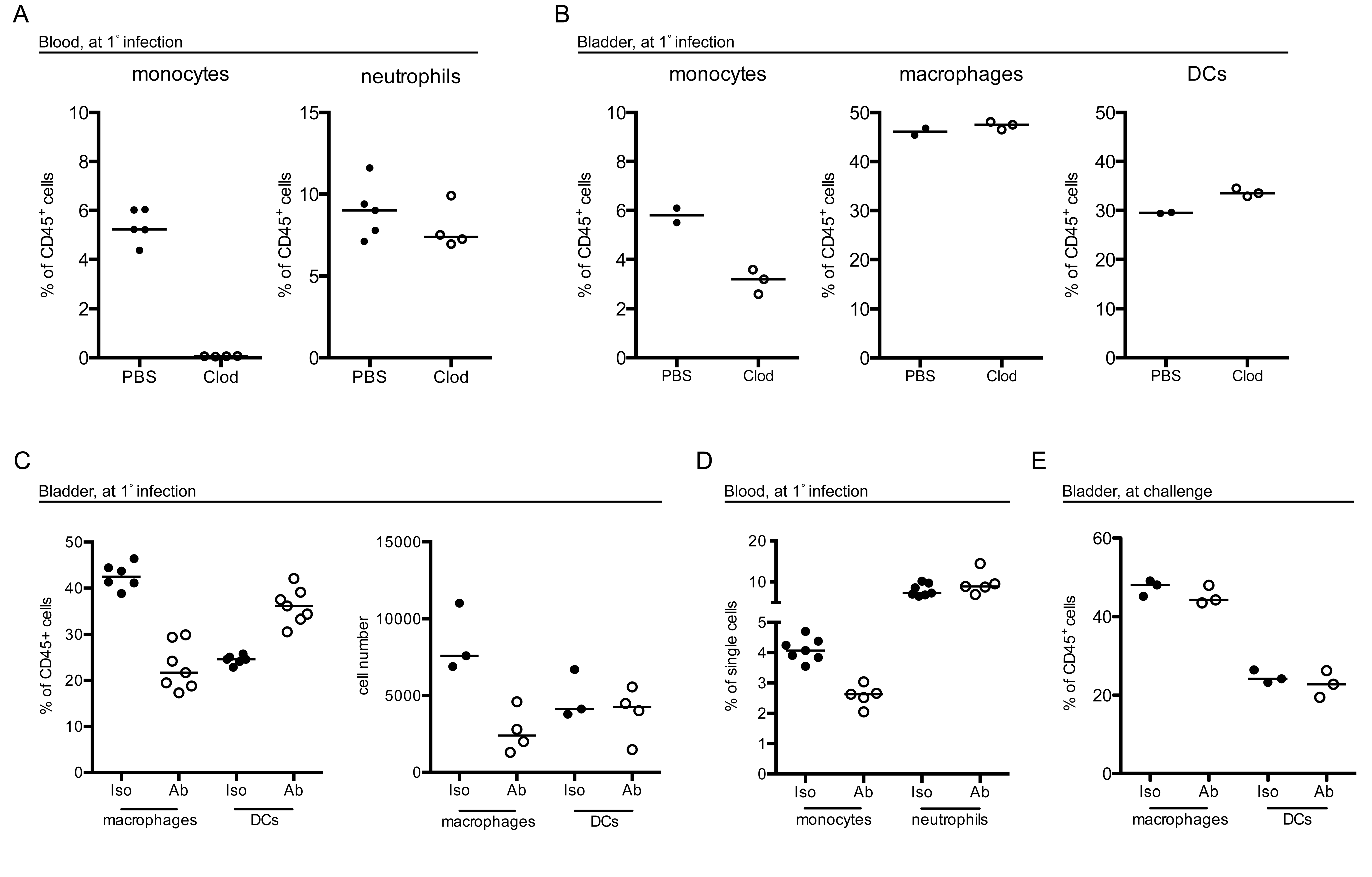

Supplement: S3 Fig — (A-B) Mice were treated with PBS or clodronate liposomes (Clod) I.V. and 15–18 hours later, blood and bladder samples were obtained to evaluate immune cell depletion. Graphs depict the percentage of (A) monocytes and neutrophils in blood and (B) monocytes, macrophages, and DCs in the bladder after treatment. (C-D) Mice received two injections of anti-CSF1R antibody (Ab) or control isotype antibody (Iso) and 24 hours post-treatment, naive bladders were isolated to evaluate immune cell depletion. Graphs show the (C) percentage and cell number of macrophages and DCs in the bladder and (D) percentage of monocytes and neutrophils in the blood. (E) Mice were depleted of macrophages as in (C-D), however, bladders were evaluated for repopulation by macrophages 4 weeks after depletion, prior to challenge infection in additional cohorts of treated mice. Each dot represents one mouse. Experiments were repeated 2–4 times with 2–7 mice per group. (TIF) [file ppat.1005044.s003.tif]

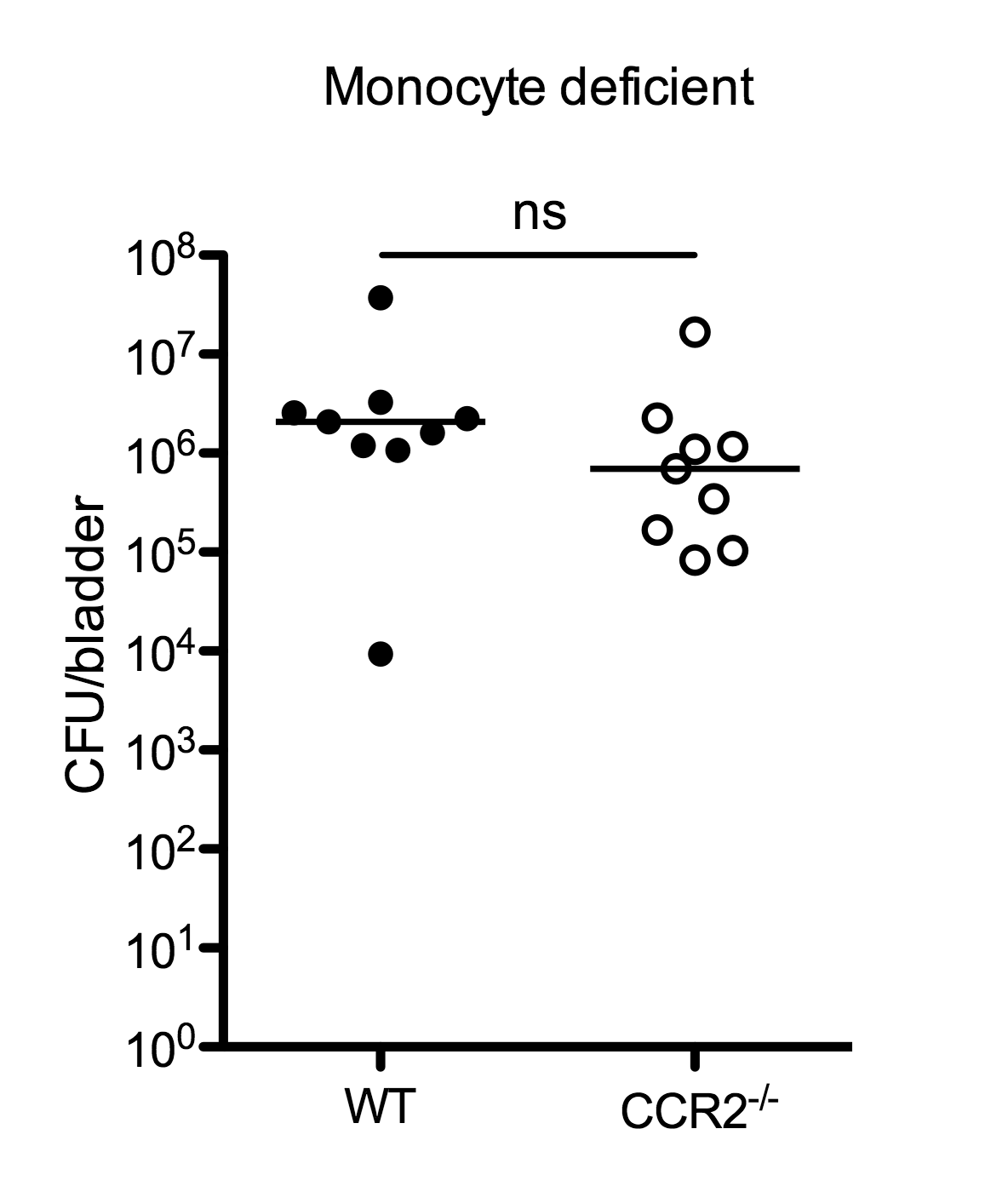

Supplement: S4 Fig — Graph depicts the CFU/bladder 24 hours post-primary infection in wildtype (WT) or CCR2-/- mice. Experiment was repeated 2 times with 4–5 mice per group. (TIFF) [file ppat.1005044.s004.tiff]

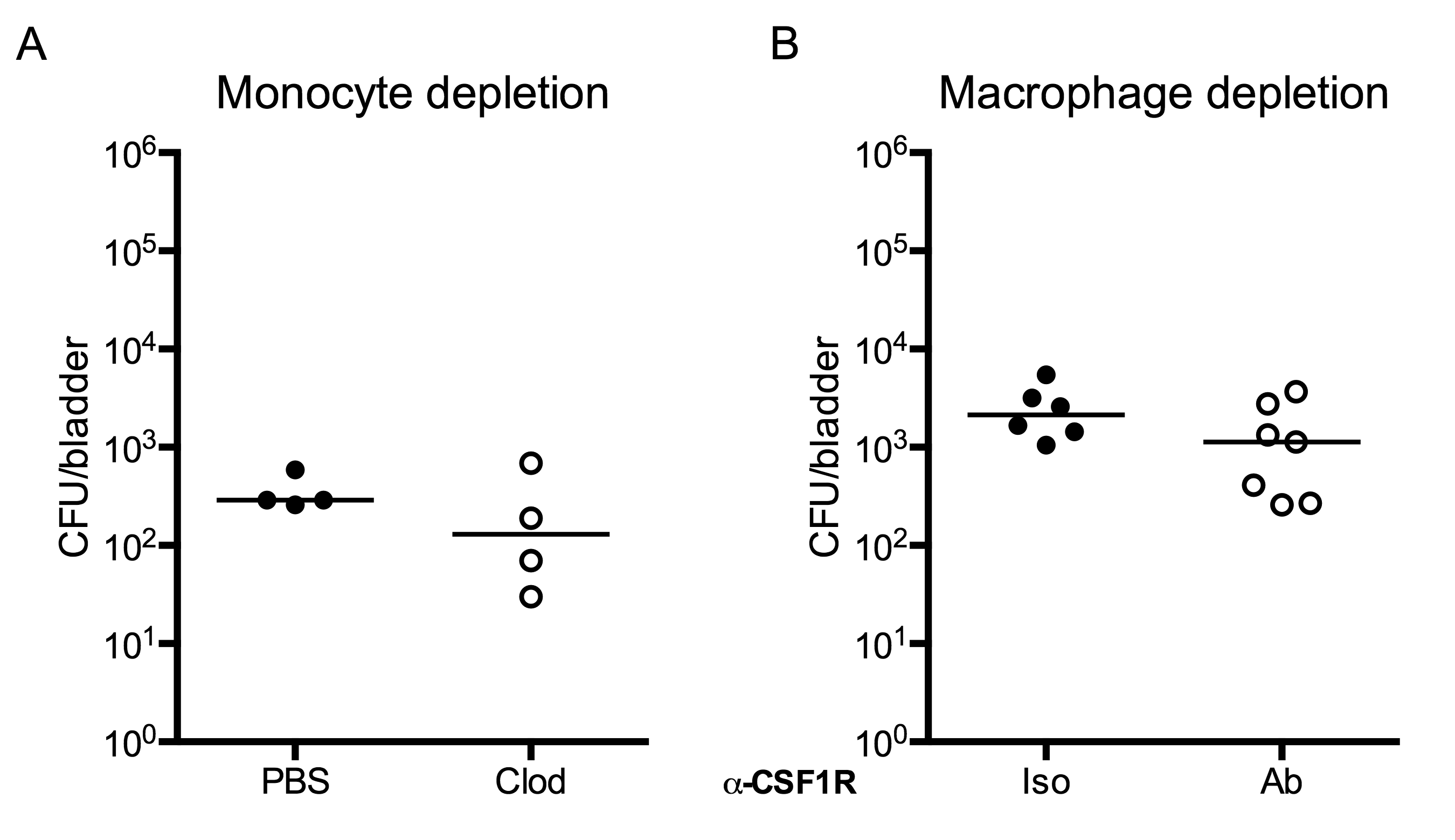

Supplement: S5 Fig — Graphs depict CFU/bladder arising from the primary infecting strain in an experiment in which (A) monocytes or (B) macrophages were depleted prior to primary infection and then challenged with an isogenic strain and sacrificed 24 hours post-challenge. Each dot represents one mouse. Experiments were repeated 2–4 times with 2–7 mice per group. (TIFF) [file ppat.1005044.s005.tiff]

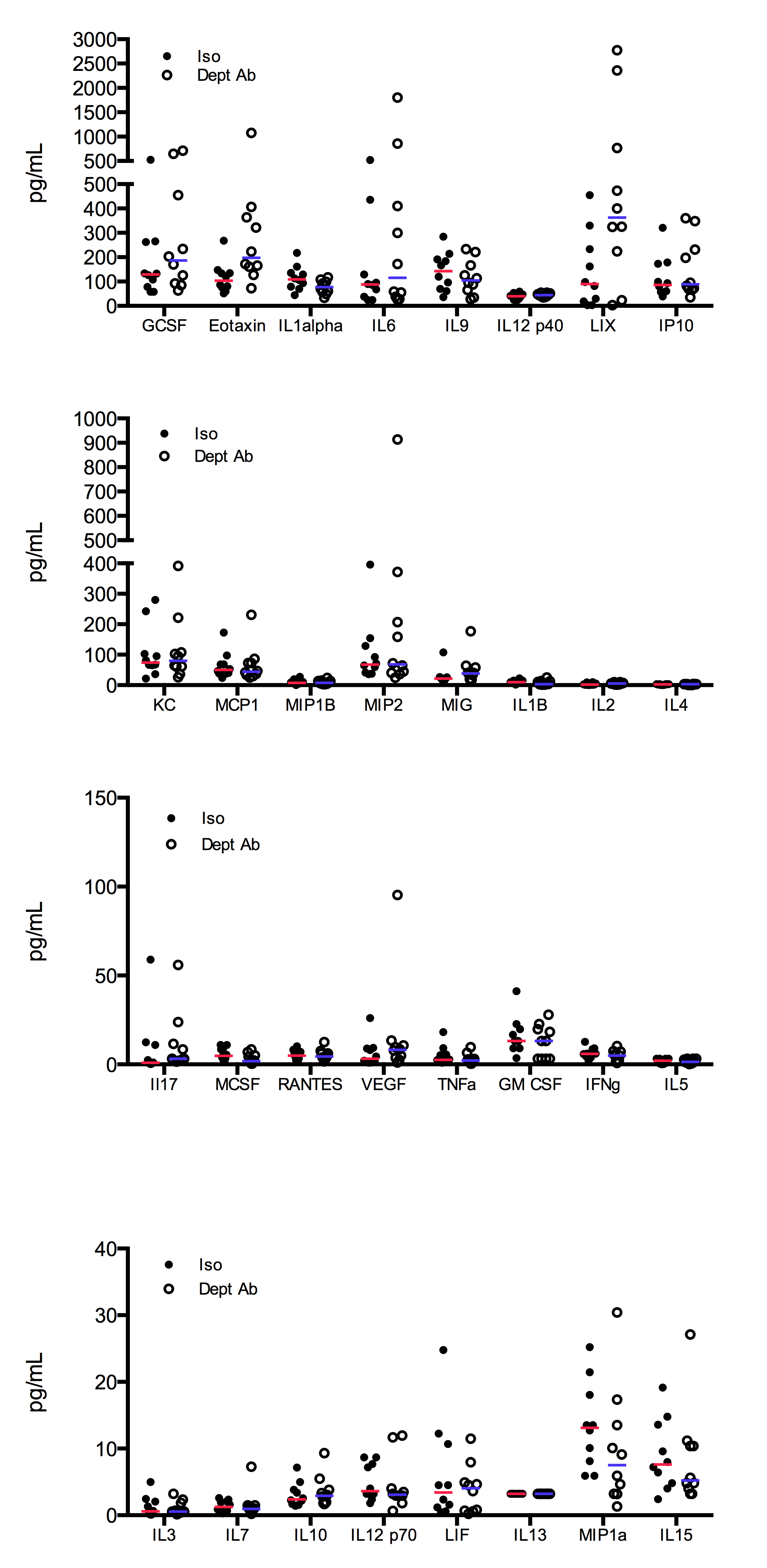

Supplement: S6 Fig — Mice were depleted with anti-CSF1R antibody and infected with 1x10^7^ CFU of UTI89 24 hours after depletion. Mice were sacrificed 24 hours P.I. and bladders were homogenized. Samples were stored at -80°C until all samples could be assessed together by Luminex multi-analyte profiling, to avoid inter-assay variability. Graphs depict the expression levels of selected cytokines in isotype antibody treated (black dots, red medians) and depleting-antibody treated (open circles, blue medians) mice. Analytes are grouped by high expression (top) to low or no expression (bottom). Each dot represents a mouse, experiment performed 2 times with 5 mice per group and all data pooled. (TIFF) [file ppat.1005044.s006.tiff]

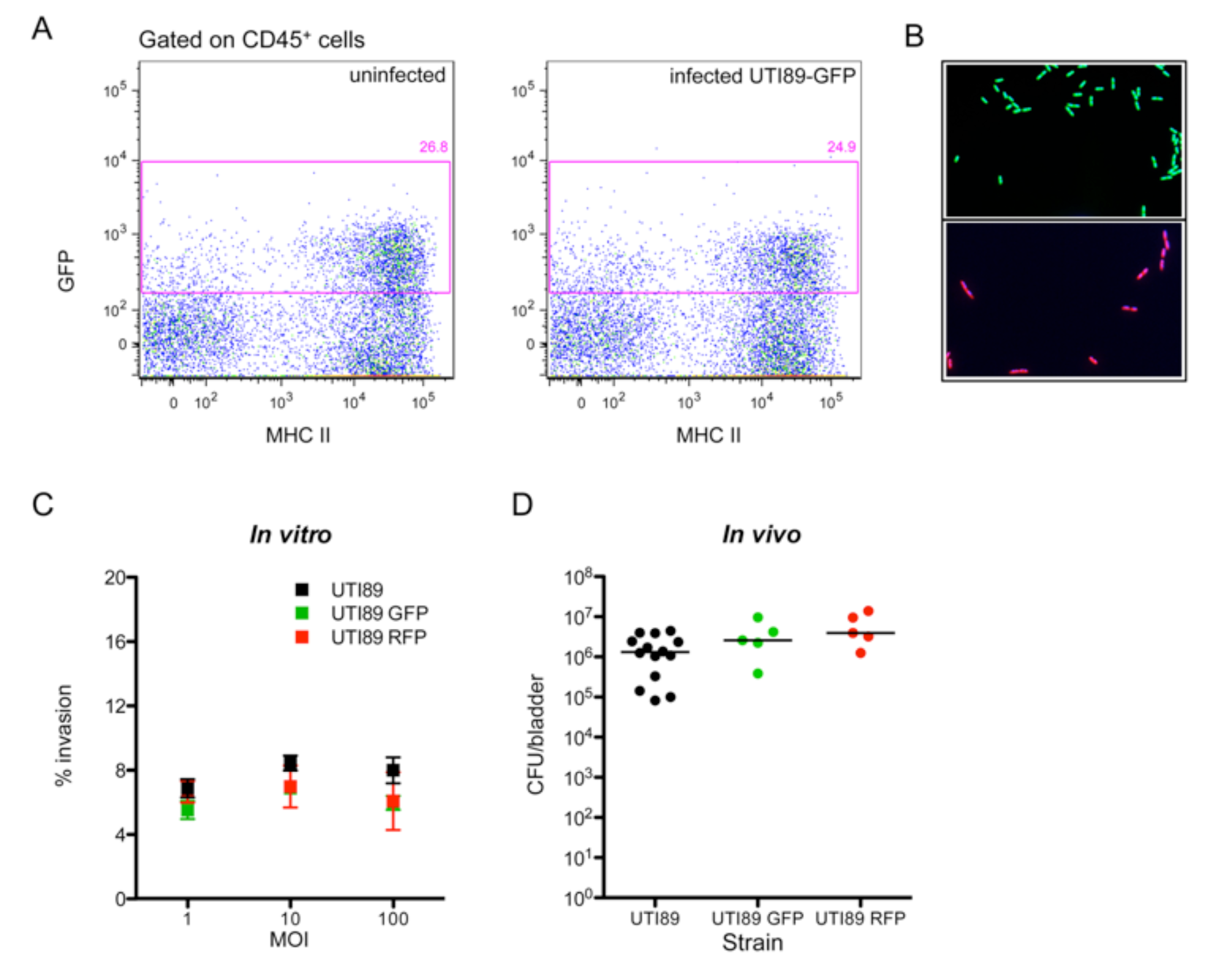

Supplement: S7 Fig — (A) Cytometry plots, gated on all CD45+ cells, depict GFP fluorescence (gated in pink with percentages) in mice either uninfected or infected with UTI89-GFP at 4 hours post-infection. (B) Fluorescence of UTI89-GFP and UTI89-marsRFP was confirmed by microscopy. (C) The mouse urothelial cell line, NUC-1, was infected with the parental UTI89, UTI89-GFP, or UTI89-RFP at an MOI of 1,10, or 100. Cells were lysed and bacterial titers determined by serial dilution 30 minutes P.I. The percentage of invasion refers to the number of bacteria obtained after infection x 100/number of bacteria in the inoculum. (D) Mice were instilled with 1x10^7^ CFU of UTI89, UTI89-GFP, or UTI89-RFP. CFU per bladder were determined by serial dilution at 24 h P.I. Each dot represents one mouse. Experiments were repeated 2 times. (TIF) [file ppat.1005044.s007.tif]
